# Sleep timing and duration in indigenous villages with and without electric lighting on Tanna Island, Vanuatu

**DOI:** 10.1038/s41598-019-53635-y

**Published:** 2019-11-21

**Authors:** Andrea N. Smit, Tanya Broesch, Jerome M. Siegel, Ralph E. Mistlberger

**Affiliations:** 1 Department of Psychology, Simon Fraser University, Burnaby, BC V5A1S6 Canada; 2 Department of Psychiatry and Biobehavioral Sciences, University of California, Los Angeles, Los Angeles, CA 90095 USA; 3 VA Greater Los Angeles Healthcare System, 16111 Plummer Street, Los Angeles, CA 91343 USA; 4 Brain Research Institute, University of California, Los Angeles, Los Angeles, CA 90095 USA

**Keywords:** Sleep, Human behaviour

## Abstract

It has been hypothesized that sleep in the industrialized world is in chronic deficit, due in part to evening light exposure, which delays sleep onset and truncates sleep depending on morning work or school schedules. If so, societies without electricity may sleep longer. However, recent studies of hunter-gatherers and pastoralists living traditional lifestyles without electricity report short sleep compared to industrialized population norms. To further explore the impact of lifestyles and electrification on sleep, we measured sleep by actigraphy in indigenous Melanesians on Tanna Island, Vanuatu, who live traditional subsistence horticultural lifestyles, in villages either with or without access to electricity. Sleep duration was long and efficiency low in both groups, compared to averages from actigraphy studies of industrialized populations. In villages with electricity, light exposure after sunset was increased, sleep onset was delayed, and nocturnal sleep duration was reduced. These effects were driven primarily by breastfeeding mothers living with electric lighting. Relatively long sleep on Tanna may reflect advantages of an environment in which food access is reliable, climate benign, and predators and significant social conflict absent. Despite exposure to outdoor light throughout the day, an effect of artificial evening light was nonetheless detectable on sleep timing and duration.

## Introduction

Sleep timing and duration in humans are determined in part by a master circadian clock entrained to local time by retinal inputs encoding environmental light-dark (LD) cycles^1^. The clock is phase delayed by light in the evening and early night, and advanced by light in the late night and early morning^2,3^. With industrialization and on-demand access to electric lighting, exposure to evening light has increased, while exposure to natural light during the day has decreased^4–6^. The expected net effect is a delay in the phase at which the circadian clock aligns with local time, and thus a delay in the timing of the circadian sleep-wake cycle^7^. Depending on an individual’s social schedule (particularly work or school start times), this may result in a significant misalignment between biological time and social time, a state known as social jetlag^8^. If sleep onset is delayed, but wake onset is fixed by the social schedule, then nocturnal sleep will be restricted. Epidemiological studies have uncovered associations between short sleep (≤~6 h) and population health, while experimental studies support a causal role for sleep restriction in metabolic and other health disorders currently described as epidemic^9–14^. This leads to conjecture that a significant portion of the population in industrialized societies may sleep less than is physiologically optimal (estimated to be 7 h for adults^14^) and that this may contribute to negative trends in population health^15–17^.

Although the logic supporting this conjecture is compelling, empirical support for the argument that industrialization has caused chronic sleep restriction is weak because information about sleep duration prior to widespread availability of electric lighting is anecdotal and based on self-report. Self-reports (retrospective or diary-based) typically overestimate sleep, compared to objective measures such as actigraphy and polysomnography^18–20^. Consequently, the degree to which sleep duration has declined with industrialization may be overestimated and is controversial. Trends over the past several decades within already industrialized societies are equivocal, with some studies showing increases^21–23^, others showing decreases^24^, and some showing no systematic change in sleep length^25,26^.

Another way to estimate the impacts of industrialization on sleep is to study sleep in indigenous communities living traditional lifestyles without electric lighting. Recently, several studies have used wrist-worn accelerometers and light sensors to examine sleep patterns in communities with little reliable access to electric lighting. The findings generated from these studies have been variable. Daily sleep duration was observed to be unexpectedly short (~5 h–6.5 h group means) in traditional hunting and gathering societies in Africa and South America (the Hadza in Tanzania, the San in Namibia, and the Tsimane in Bolivia)^27,28^, an agrarian society in Madagasgar (the Malagasy)^29^, and a pastoralist society in Namibia (the Himba)^30^. By contrast, sleep duration was comparatively long in a traditional horticultural society of Papa New Guinea (~8.4 h)^31^, and in an Argentinian society (the Toba/Qom) who were traditionally hunter-gatherers (but are now mainly state supported^32^) and showed a marked seasonal variation (~7 h in summer and 8.5 h in winter)^33^. In cases where societies are transitioning to electricity, groups with access to on-demand artificial lighting showed a delay in nocturnal sleep timing compared to groups without access (Toba/Qom^33^; rural Mozambique^34^; rural Brazil^4,35,36^). In some^4,33,35^, but not all cases^34^, delayed sleep was associated with reduced sleep duration.

These results indicate that lifestyle may be an important determinant of habitual sleep duration and provide evidence for an effect of on-demand electric lighting on sleep timing and duration. To further examine the impact of lifestyle and electric lighting on sleep, we used actigraphy to measure sleep timing and duration in indigenous Ni-Vanuatu living traditional, small-scale subsistence lifestyles on Tanna Island, Vanuatu, in south pacific Melanesia. This study population provides some unique advantages, including homogeneity of ethnicity and lifestyle on the island, little seasonal variation in climate and daylength, and the availability of an electric grid in coastal but not inland villages, permitting a within-society comparison of sleep with and without access to on-demand electric lighting. Also, the latitude of Tanna Island, and thus the annual variation in photoperiod, is very close to the latitude of several hunter-gatherer societies previously shown to exhibit short sleep^27,30^, thus permitting cross-cultural comparisons with a natural control for daylength.

## Methods

### Study population

We recruited 91 adults living on Tanna Island (19.53°S, 169.27°E) to participate in our study. Forty-five of the participants lived in coastal villages with on-demand access to electricity, and 46 participants lived in villages that were up to 10 km inland and beyond the electric grid. Some data were lost due to equipment failure (*n* = 4) or were excluded due to non-compliance (i.e. extended watch removal; *n* = 5), leaving final sample sizes of 39 coastal participants (herein designated the ‘electric’ group) and 43 inland participants (‘non-electric’ group). Participants in both communities live similar lifestyles and rely primarily on small-scale farming for a livelihood. Coastal participants (electric group) are more likely to also participate in marine foraging and fishing.

Data were collected from males, females, and females who were currently breastfeeding. It was expected that breastfeeding would lead to higher levels of sleep disruption due to mother-infant co-arousal, therefore breastfeeding females (with infants <10 months of age) were maintained as a separate sample. Other females were not breastfeeding at the time of data collection and the ages of their children (if any) were over 2 years. Because fathers on Tanna Island typically take a less active role than the mother in infant rearing, it was deemed acceptable to collect males as one homogenous group irrespective of “father” status. Age and birthdays are not commonly tracked, so when documentation of age was unavailable, participant ages were estimated visually or relative to the birth of peers. Of those included in the final analysis, demographic variables and other sample characteristics are provided in Table 1.

**Table 1:** Sample characteristics by community and adult type (group means ± SD).

	Electric Communities				Non-Electric Communities			
	Males	Females	BrF. Females	Total	Males	Females	BrF. Females	Total
*n*	13	13	13	**39**	15	13	15	**43**
Est. Age (yrs)	38.3 (9.5)	33.2 (7.5)	29.1 (6.3)	**33.54 (8.57)**	36.3 (9.3)	34.9 (8.7)	31.4 (8.0)	**34.12 (8.74)**
Age range	21–48	24–49	20–43	**20–49**	22–53	22–46	21–43	**21–53**
Height (ft)	5.62 (0.19)	5.42 (0.16)	5.28 (0.20)	**5.44 (0.23)**	5.57 (0.24)	5.43 (0.21)	5.04 (0.41)	**5.34 (0.38)**
Weight (lbs)	170.8 (23.6)	148.4 (25.0)	148.3 (22.4)	**155.86 (25.49)**	134.7 (30.6)	138.3 (17.1)	129.3 (21.9)	**133.82 (23.79)**
Body Fat %	21.15 (7.35)	30.85 (7.19)	31.08 (6.60)	**27.69 (8.31)**	15.50 (4.77)	28.33 (7.23)	25.47 (6.27)	**22.90 (8.12)**
% Married	92.3%	69.2%	69.2%	**79.0%**	86.7%	69.2%	86.6%	**81.4%**
% Sleep with Partner	91.7%	66.7%	44.4%	**72.4%**	100%	77.8%	92.3%	**91.4%**
% Have Children	76.9%	85.2%	100%	**84.6%**	93.3%	92.3%	100%	**95.4%**
Avg # Children	3.20	2.46	2.54	**2.70**	3.79	3.16	4.20	**3.76**
Est. Age Children (yrs)	9.85	10.76	5.56	**8.56**	8.07	9.87	7.09	**7.81**
# Co-habitating	4.10	3.15	4.15	**3.77**	4.33	4.07	5.00	**4.48**
# Co-sleepers	4.92	3.23	3.61	**3.55**	5.00	4.15	5.09	**4.53**

Note: BrF. Females = Breastfeeding females. Est. Age is the estimated average age (age & birthdays are not tracked). Weight & Body fat % were measured using a Tanita UM-028F Scale. All other data are subjective and collected through interview. # Cohabitating & # Co-sleepers represents the average total number of individuals (adults & children) living together or sharing a sleeping space (includes individual interviewed).

We obtained research permits from the Vanuatu Cultural Centre as well as permission from the elders and chiefs in the host communities. Participants were recruited by word of mouth. We explained the details of the study and obtained informed consent verbally from each participant, as outlined by the Office of Research Ethics at Simon Fraser University, Burnaby BC, Canada. Gifts equivalent to $5 CAD were given for participation. All procedures were performed in accordance with relevant guidelines and regulations and approved by Simon Fraser University.

### Light at night

**Figure 1:**
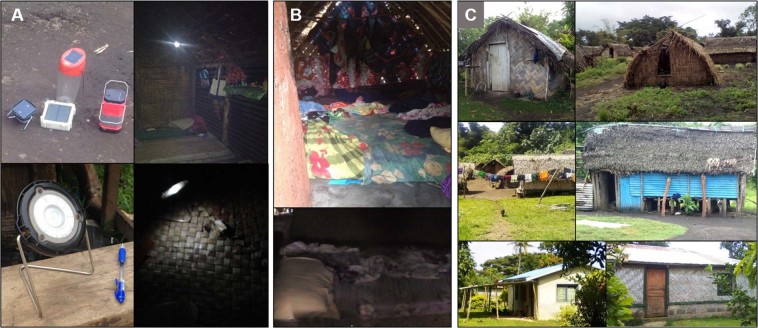
Lifestyle images; Panel (A) Solar light sources in non-electric villages; Panel (B) Sleeping spaces in non-electric (upper) and electric (lower) villages; Panel (C) Dwelling types found in both electric and non-electric villages, with natural material ‘grass huts’ being most common (upper & middle left = grass huts; middle right = corrugated tin hut; lower = huts incorporating cement as foundation or walls, with tin roofs).

Sources of light at night were most often a singular incandescent lightbulb inside dwellings powered by electrical grid (herein referred to as electric light), and/or small solar powered LED lights (Fig. 1A; herein referred to as ‘solar torches’). Solar torches were placed on the floor to facilitate household duties or were carried by hand when walking through the village. Light intensity provided by the torches did not exceed 2 lux, measured at 1-meter distance using the Actiwatch-2 light sensor (Phillips Respironics, Murrysville, PA). During the dates of this study, all participants from communities with access to electricity reported using artificial lights. Although seven participants living in electric communities did not have working electricity at the time of data collection, they each used solar torches, and reported exposure to electric light. Most of the participants from villages without electricity either owned or shared solar torches for use at night, and daily evening use was reported by 84% of participants in non-electric communities. Electronic devices were almost non-existent with the exception of some basic mobile phones, which were not a common source of light at night, especially in villages off the electric grid as there was no ready access to charging stations.

### Sleeping spaces

Sleeping arrangements were variable, but most sleeping spaces consisted of blankets or foam mattresses on grass woven mats on the floor of the dwelling (Fig. 1B). Floors were commonly wood plank, or hard packed ground. Co-sleeping is typical on Tanna Island; all participants in this study shared sleeping quarters with multiple children or adults. Sleeping spaces were shared by immediate family (i.e. parents and children), and sometimes with extended family as well (Table 1). Sleeping arrangements are often flexible and can change readily. Traditional homes are made of local natural materials, (e.g., palm leaves, bamboo) carefully constructed to withstand cyclones Separate dwellings are used for sleeping and cooking. A few dwellings incorporated cement or tin components (Fig. 1C). Little time is spent inside during the day.

Temperature and humidity in typical sleeping spaces were measured with iButtons (Maxim Integrated, San Jose, CA) sampling at 20-minute intervals throughout the study interval. The iButtons were placed in representative huts in one electric village and one non-electric village. Temperature in the non-electric village during this period averaged ~24.6 °C (86% RH) in the day and ~22.9 °C (88% RH) at night, with an average daily range of 3.9 ± 1.8 °C. Electric villages were slightly warmer and drier, averaging ~25.9 °C (79% RH) in the day and ~23.9 °C (80% RH) at night, with a daily range of 3.6 ± 1.8 °C. The daily temperature minimum occurred at ~06:20 h, and the maximum between 13:20–14:00 h, in both communities.

### Lifestyle

Although coffee beans are exported from Tanna Island, caffeinated beverages are not commonly used or readily available in the villages studied. All men drink a beverage of kava root, which, although only a few of its constituents have been studied, has been found to have mild sedative, anxiolytic, and antinociceptive/analgesic properties^37–39^. Sixty-five percent of male participants reported drinking kava on a daily basis. Women are not traditionally permitted to drink kava for leisure nor are they permitted to participate in Kava ceremonies. Food consists primarily of locally cultivated foods, such as root vegetables (taro, yam, sweet potato, manioc), seasonal fruit (plantain, oranges, grapefruit, coconut), and on occasion purchased rice, and chicken, beef, fish or pork for ceremonies/celebrations. Cooking takes place over a fire, or with hot stones in an earth oven cooking pit. Breakfast and dinner times were regimented but flexible, and men who attended the nakamals would often eat late. Lunch times were not regimented .

Although both community types practice primarily subsistence horticultural lifestyles, 23% of the population of each community type report spending some time participating in wage labour. Farming is the primary daily activity for 93% of the non-electric community members compared to 41% of the electric community members, who report spending more time on other daily activities within the village (i.e., fishing, building). Most women (including females with and without small infants) report their primary evening activity to be caring for children (85% with electricity, 93% without) and attendance at church groups, whereas men report spending time in the nakamal (gathering place) for kava drinking (77% with electricity, 73% without).

Bislama is the national language in Vanuatu, but many distinct indigenous oral languages exist on Tanna, and can vary even between nearby villages. Although some terms exist to refer to times of the solar day, residents of Tanna Island do not quantitatively track time within a day (i.e. no clocks, sun dials, etc.), and there are no designated work and free days. Alarm clocks were not used by the participants of this study.  Chickens and small pigs wander freely through the villages and often alert residents to sunrise. Individuals do report taking days off from agrarian responsibilities, but working in the garden on these days is replaced with obligations for religious worship (e.g. church attendance). Formal education is not common, but is increasing in prevalence, especially for villages in close proximity to a school, which is the case for electric villages. Morning social obligations included early awakening by some women to prepare children for school (reported by 5 women in electric, and 1 in non-electric communities), or to travel to Wednesday market to sell produce to make money for school fees. We did not collect information on the prevalence of school attendance but as noted, electric villages where closer to schools or to roads where vehicles travel.

### Procedure

Data were collected over a period of 3 weeks between April 14 and May 8, 2017. On the first day of recording, sunrise occurred at 05:52 h and sunset at 17:32 h. By the last day of recording, sunrise had delayed 8 minutes to 06:00 h, and sunset had advanced 15 minutes to 17:17 h [Source: National Research Council of Canada; http://app.hia-iha.nrc-cnrc.gc.ca/cgi-bin/sun-soleil.pl]. Based on distance, sunrise and sunset should have differed by no more than 1 minute between community types.

Participants were asked to wear an Actiwatch-2 activity monitor on their wrist for 7 days. These devices use accelerometers to measure movement at 32 hz, which was binned into 15 second epochs. Participants were instructed not to remove the watch, but abrupt periods of inactivity with invariant or no light level were evident in some records.

Participants were visited during data collection week to complete an interview about lifestyle habits and use of electric light (see Supplementary Materials). The interviews were administered by trained local translators and were conducted in the indigenous language specific to each village. Body weight, body fat % (using a Tanita UM-028F scale) and height were recorded. Participants also completed a follow-up questionnaire on day 7, where they indicated if there was anything that had disrupted their sleep from its usual pattern (e.g., illness, celebration, etc.). This information was used primarily to inform Actiwatch data cleaning (e.g., exclusion of nights in which a participant was sick), and to qualitatively understand aspects of local lifestyles that disrupt sleep.

### Actiwatch data cleaning

Sleep and wake states were scored by Actiware 6.0.9 software (Phillips Respironics, Murrysville, PA) using the default settings, except in the scenarios below. The software uses movement data to score behavioral state as wake, rest and sleep. Rest bouts typically occur immediately before and after sleep bouts, and were used to define bedtime and rise time. Subjects were anonymized and each actogram was visually inspected prior to analysis.

#### Exclusions

Segments within a day were excluded if (a) the participant indicated during interview or follow-up that they had removed the watch, or if the participant was observed without the watch, or; (b) if no activity counts were registered for >2 hours and the participant did not report napping at this time. Thirty minutes of consistent activity determined the point at which data would again be included for analysis. If >4 hours (1/6 of the day) were excluded, then activity and light counts for that entire 24 h recorded day were excluded. In such cases, sleep onset and wake events were analyzed for those days that were available.

#### Sleep fragmentation removal

**Figure 2:**
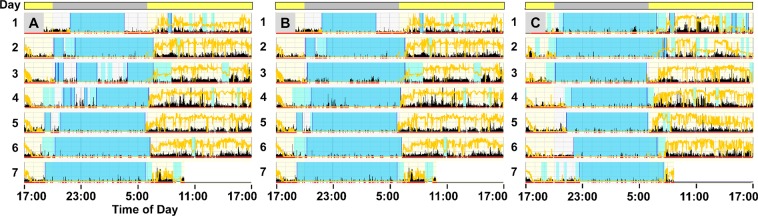
Representative Actograms from two males in the electric community. Periods of sleep are blue, activity is shown as black ticks, light exposure is yellow, consecutive days are stacked vertically. Panel (A) Fragmented nocturnal sleep (interrupted blue sections), of which, night 3 and 4 qualified for fragmentation removal (see Methods, Data Cleaning); Panel (B) The same subject with nocturnal sleep periods following manual fragmentation removal; note that combining the fragmented sleep periods did not change sleep duration since wake during the night (black ticks) was subtracted from overall sleep duration. Panel (C) Naturally consolidated nocturnal sleep in another subject.

In 73 of the 519 nights included in analysis (14% of cases), nocturnal bouts of activity were detected by actimetry that were sufficient to divide nocturnal sleep into two bouts (e.g., Fig. 2). In these cases, Actiware software chose sleep onset and wake times from the longer of the two sleep bouts to represent the sleep period for that night. If the following criteria were met, then the two sleep bouts were manually joined to allow reported sleep onset and wake time to represent one sleep period across the entire nocturnal period (by using sleep onset from the earlier bout and wake time from the later bout). Combining the two sleep periods did not change total nocturnal sleep duration, as any periods of awakening during the night were not included as sleep. The combining procedure served to consolidate the sleep bouts into one sleep period with reduced sleep efficiency. Criteria for combining sleep periods were, (a) the period of nocturnal activity must have occurred during a time when the subject was “usually” asleep/inactive (based on visual verification from other nights in the actogram), (b) the subject must have been asleep for at least 2 h prior to the period of awakening, or (c) the period of nocturnal awakening had to be shorter than the shortest period of sleep. For example, if a subject slept for 30 minutes, awoke for 2 h and went back to sleep for 6 h, this would not qualify for fragmentation removal, and the 6 h sleep period would be considered their nocturnal sleep period. However, if a subject slept for 4 h, awoke for 2 h, and then slept for 4 h, this would qualify for fragmentation removal (therefore nocturnal sleep duration would be 8 h, and the sleep period would be 10 h).

### Actiwatch analyses & statistics

Dependent variables were calculated as follows. Raw light and activity counts from the Actiwatches were averaged across subjects. Actiware 6.0.9 software was used to score bedtime, sleep onset, wake time, rise time, nocturnal sleep duration (the time in minutes between sleep onset and wake time minus the number of minutes awake after sleep onset), sleep efficiency (number of minutes awake after sleep onset divided by the number of minutes between sleep onset and wake time), and nap duration. Nocturnal sleep duration and nap duration were summed to yield 24 h total sleep time (TST). Activity data were imported into Clocklab 6.0 (Actimetrics, IL) in 1 min bins for calculation of non-parametric circadian variables, including L5 (mean activity during the 5 least active consecutive hours each 24 h day), M10 (mean activity during the 10 most active consecutive hours), relative amplitude (RA; the ratio of M10-L5 to M10 + L5), intradaily variability (IV; the number of transitions between rest and activity bouts per day) and interdaily stability (IS; the variability in the timing of rest and activity bouts across days).

JMP 14 (SAS Institute, Cary, NC) and Prism 7.0 (GraphPad Software Inc., San Diego) were used for inferential statistics and to produce figures. The primary aim was to assess the relationships between sets of independent variables (community type and adult type) and dependent variables (i.e. sleep timing, nocturnal sleep duration, sleep efficiency) using separate analyses of variance (ANOVA) for each dependent variable. Prior to conducting these ANOVAs, we explored the effects of potential covariates, such as age, number of co-sleepers, and body fat percentage by observing whether the potential covariates significantly correlated with the various dependent variables. The only significant correlation observed was between age and sleep efficiency. Accordingly, to assess the relationships among community type (electric, non-electric) and adult type (males, females, breastfeeding females) with sleep timing variables and sleep duration variables, separate 2 × 3 ANOVAs were conducted for each dependent variable. To account for the significant relationship between age and sleep efficiency, a (2 × 3) ANCOVA was used to emulate the ANOVAs, but with age entered into the model as a covariate. Tukey’s post hoc tests were used to further explore significant main effects of adult type, or significant interactions. In cases where parametric tests were not appropriate (i.e. violation of assumption of homogeneity of variance; identified using Welch’s Test), Mann-Whitney U non-parametric independent samples tests were performed between community types, foregoing analysis by adult type. Statistical significance was defined at p < 0.05 (two-tailed, unless otherwise stated due to a priori directional hypotheses). Figures including means are plotted ± standard error of the mean (SEM).

## Results

### Activity

**Figure 3:**
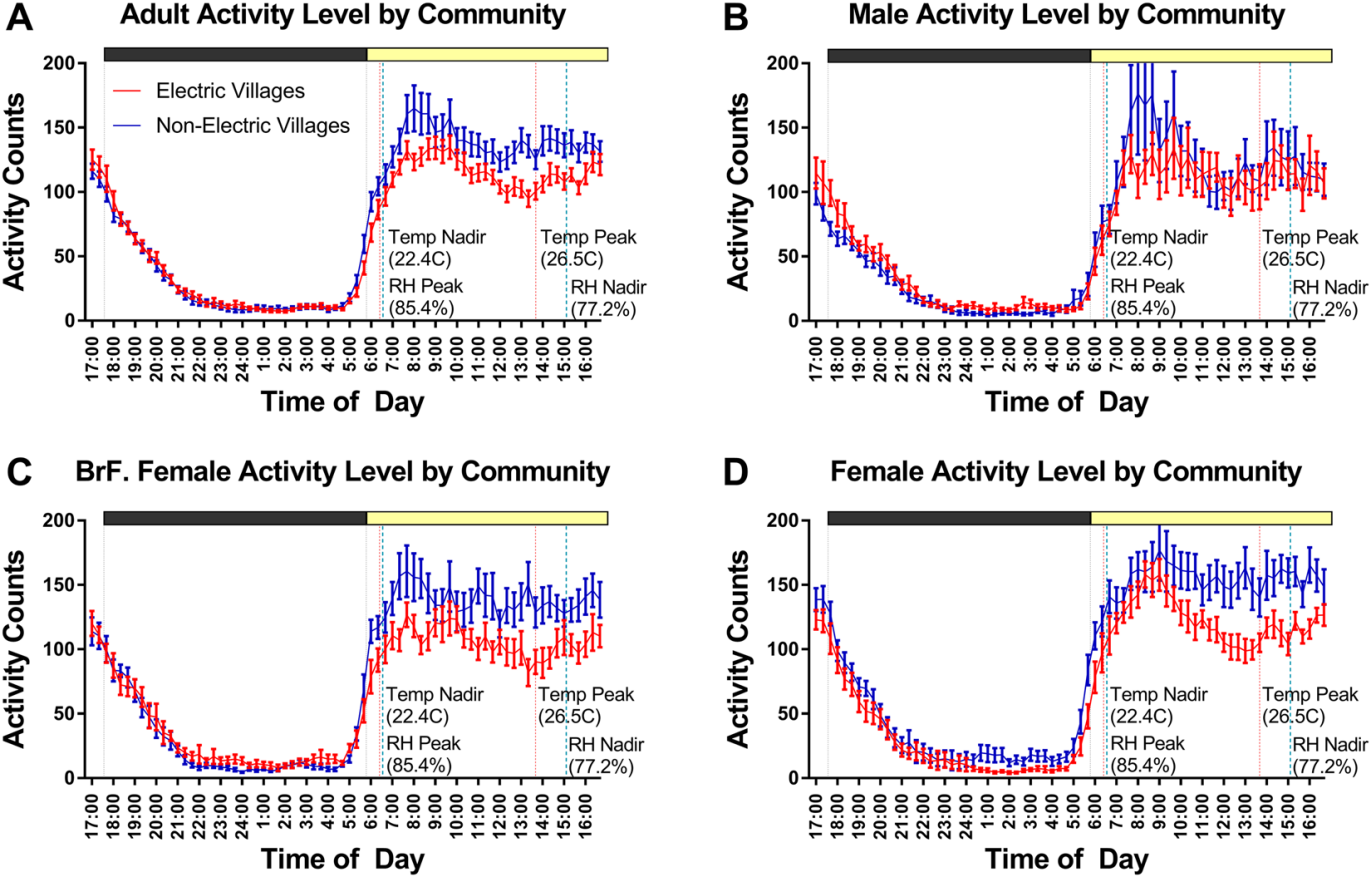
Group mean (±standard error) waveforms of activity in 20-minute time bins, comparing community type (Panel (A) electric in red; non-electric in blue) for each adult category (Panel (B) males; Panel (C) breastfeeding females; Panel (D) females). Vertical lines indicate day/night transition (sunrise & sunset; grey), and environmental temperature (degrees Celsius; pink) and relative humidity (RH %; light blue) peak and nadir.

Actiwatch activity data were averaged in 20-min bins for each subject and then plotted as group mean waveforms (Fig. 3). During the daytime, activity levels among residents of the non-electric communities were higher than among residents of the electric communities (Fig. 3A), but differences between groups in the 10 most active hours in the day (M10) did not meet the threshold for statistical significance (median electric = 461.1, n = 39; median non-electric = 507.8, *n* = 43; Mann-Whitney *U* = 641, *p* = 0.067). M10 onset time was slightly later in the electric group, but again this fell just below the chosen cutoff for statistical significance (median electric = 07:19 h, *n* = 39; median non-electric = 06:52 h, n = 43; Mann-Whitney *U* = 774, *p* = 0.055).

The 5 least active consecutive hours of the night (L5) did not differ between community types in level (median electric = 24.8, n = 39; median non-electric = 22.8, n = 43; Mann-Whitney *U* = 723, *p* = 0.287) or time of onset (median electric = 23:02 h, n = 39; median non-electric = 23:05 h, n = 43; Mann-Whitney *U* = 730.5, *p* = 0.32). Relative amplitude also did not differ significantly between groups (median electric = 0.90, *n* = 39; median non-electric = 0.91, *n* = 43; Mann-Whitney *U* = 690, *p* = 0.170).

Intradaily variability (IV) represents the degree of fragmentation of rest-activity patterns, and was significantly greater in the electric communities (*F*_(1,76)_ = 11.83, *p* < 0.001). Interdaily stability (IS) represents the degree of synchrony to the 24 h light-dark cycle and did not differ by community type (*F*_(1,76)_ = 0.15, *p* = 0.678). There was a significant main effect of adult type for both IV (*F*_(2,76)_ = 11.47, *p* < 0.001) and IS (*F*_(2,76)_ = 3.17, *p* = 0.048), but no significant interactions for either IV (*F*_(2,76)_ = 0.18, *p* = 0.832) or IS (*F*_(2,76)_ = 0.85, *p* = 0.43). Numerically, breastfeeding females had the highest levels of both IV and IS (Table 2) compared to other adult type groups.

**Table 2:** Sleep parameters by community and adult type (means ± SD).

	Electric Communities				Non-Electric Communities				Significance value between Communities
	Males	Females	BrF. Females	Total	Males	Female	BrF. Females	Total	
*n*	13	13	13	**39**	15	13	15	**43**	
Bedtime (h:mm)	21:16* (0:41)	20:32 (0:56)	20:44 (0:47)	20:50* (0:51)	20:54 (0:59)	20:44 (1:02)	20:03 (0:32)	**20:33 (0:57)**	*p* = 0.138
Sleep Onset (h:mm)	21:42* (0:38)	21:02 (0:59)	21:14 (0:49)	21:19* (0:51)	21:19 (0:56)	21:05 (0:58)	20:24 (0:36)	**20:56 (0:55)**	*p* = 0.022
Wake Up (h:mm)	6:20 (0:46)	6:04 (0:57)	5:20 (0:33)	**5:54 (0:52)**	6:17 (1:05)	5:36 (0:47)	5:26 (0:24)	**5:47 (0:52)**	*p* = 0.467
Rise Time (h:mm)	6:39 (0:43)	6:19 (1:00)	5:36 (0:31)	**6:11 (0:53)**	6:37 (1:05)	5:53 (0:50)	5:44 (0:23)	**6:05 (0:54)**	*p* = 0.533
Nocturnal Sleep Duration (hours)	7.42 (0.72)	7.91 (0.97)	6.94 (1.05)	**7.42 (1.00)**	8.05 (1.32)	7.53 (0.47)	8.02 (0.62)	**7.88 (0.92)**	*p* = 0.029
Nocturnal Sleep Efficiency (%)	79. (3.35)	83.47 (4.54)	77.80 (6.24)	**80.25 (5.31)**	82.95 (5.07)	82.53 (4.04)	82.66 (3.64)	**82.72 (4.20)**	*p* = 0.017
24 h Total Sleep Time (hours)	7.47 (0.87)	8.15 (1.05)	7.32 (1.12)	**7.65 (1.06)**	8.24 (1.16)	7.63 (0.47)	8.13 (0.66)	**8.02 (0.81)**	*p* = 0.084
Avg # Naps/Day	0.63 (0.47)	0.51 (0.40)	1.15 (0.90)	**0.77 (0.69)**	0.56 (0.54)	0.24 (0.32)	0.29 (0.35)	**0.36 (0.46)**	*p* = 0.001
Nap Duration (mins)	28.01 (9.77)	30.61 (15.18)	23.53 (7.75)	**27.27 (11.30)**	37.34 (21.53)	27.74 (15.94)	24.01 (8.60)	**32.75 (3.51)**	*p* = 0.493
Activity level: most active 10 h period (M10)	461.36 (171.88)	507.34 (76.62)	454.23 (144.22)	**474.31 (132.96)**	501.31 (245.93)	627.76 (160.74)	545.59 (172.85)	**554.98 (200.46)**	*p* = 0.067
Activity level: least active 5 h period (L5)	21.31 (12.66)	50.18 (54.12)	25.48 (9.37)	**31.37 (18.36)**	33.75 (20.80)	21.91 (14.93)	38.45 (68.20)	**31.49 (31.47)**	*p* = 0.287
Relative Amplitude	0.84 (0.43)	0.92 (0.22)	0.84 (0.25)	**0.87 (0.25)**	0.90 (0.31)	0.85 (0.58)	0.91 (0.15)	**0.89 (0.20)**	*p* = 0.170
Intradaily Variability (IV)	0.7180 (0.14)	0.6350 (0.11)	0.8329 (0.14)	**0.7286 (0.19)**	0.6273 (0.15)	0.5425 (0.14)	0.7025 (0.12)	**0.6279 (0.20)**	*p* < 0.001
Interdaily Stability (IS)	0.5194 (0.14)	0.5772 (0.11)	0.6212 (0.07)	**0.5726 (0.12)**	0.5749 (0.19)	0.5167* (0.07)	0.6402 (0.08)	0.5987* (0.13)	*p* = 0.678
Daytime Light Avg Lux (6:00–17:20)	1958.46 (753.59)	1812.37 (633.99)	1169.52 (539.93)	**1747 (919)**	2674.65 (1144.59)	1964.22 (1284.54)	1810.13 (573.10)	**2158 (1081)**	*p* = 0.068
Evening Lux (18:00–0:00)	0.12 (0.11)	0.22 (0.25)	0.31 (0.43)	**0.33 (0.43)**	0.10 (0.10)	0.14 (0.15)	0.04 (0.02)	**0.15 (0.19)**	*p* = 0.016
Morning Lux (3:00–5:20)	0.05 (0.06)	0.05 (0.07)	0.13 (0.17)	**0.07 (0.11)**	0.02 (0.01)	0.04 (0.05)	0.02 (0.01)	**0.02 (0.03)**	*p* = 0.171

Note: *indicates outlier removal (n −1).

### Light

**Figure 4:**
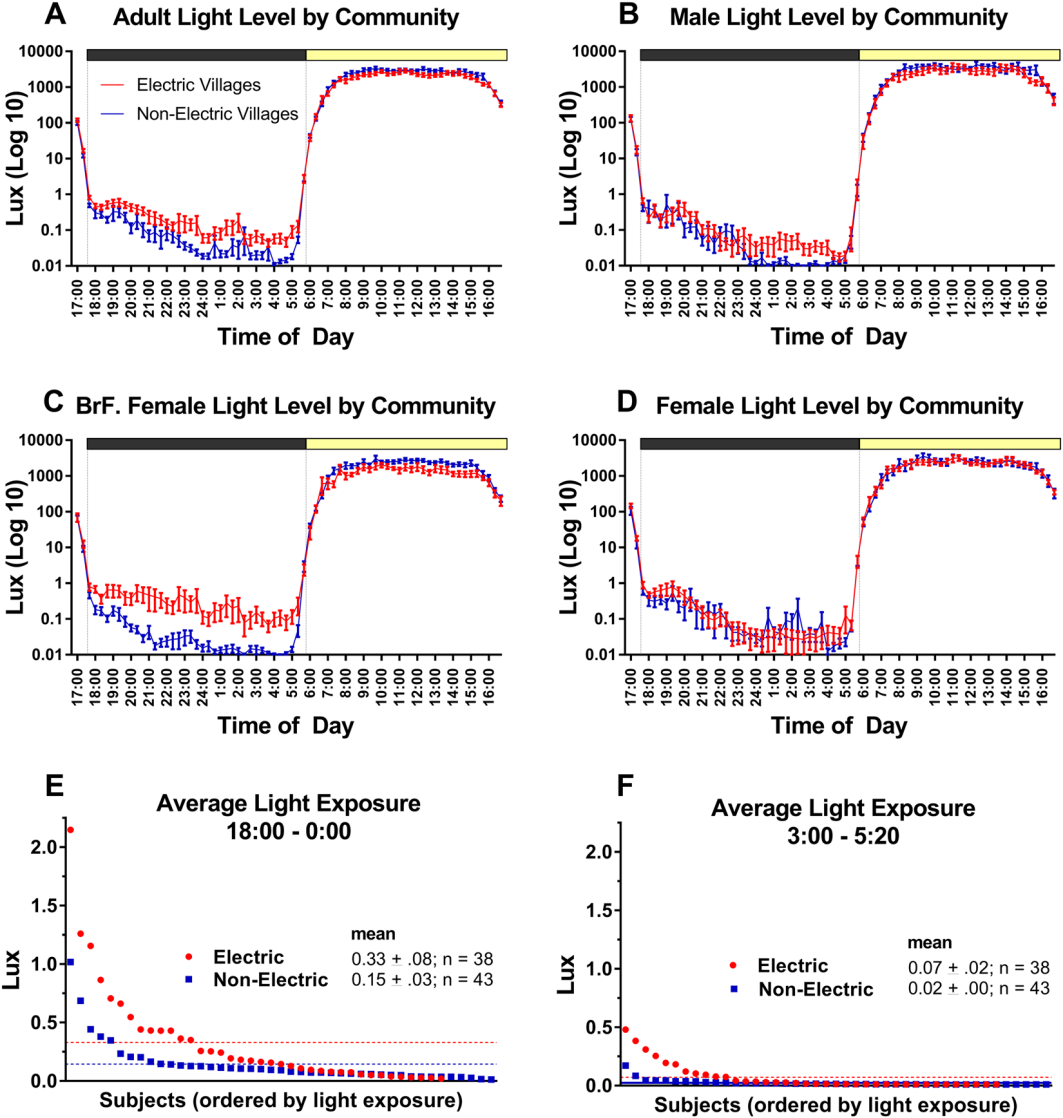
Panel (A–D) show group mean (±standard error) waveforms of light exposure in 20-minute time bins, comparing community type (Panel (A) electric in red; non-electric in blue) for each adult category (Panel (B) males; Panel (C) breastfeeding females; Panel (D) females). Vertical grey line indicates day/night transition (sunrise & sunset). Panel E and F show average light exposure (lux) measured by Actiwatch-2 for subjects in post-sunset (18:00–0:00) and pre-sunrise (3:00–5:20) hours (presumably corresponding to times when light would lead to phase delays and advances, respectively). Horizontal dashed lines indicate mean light exposure for electric (red) and non-electric (blue) communities, corresponding to numerical means presented (±standard error).

Actiwatch light exposure data were averaged in 20-min bins for each subject, and then plotted as group mean waveforms (Fig. 4). During the study month, sunrise occurred on average at 05:56 h (average civil twilight start = 05:33 h), and sunset at 17:23 h (average civil twilight end = 17:46 h). During the daytime (06:00 h – 17:20 h), both communities were exposed to similar amounts of light throughout the day (median electric = 1550 lux, n = 38; median non-electric = 1721 lux, n = 43, Mann-Whitney U = 624, p = 0.068). Peak illuminance averaged 2894 ± 2029 lux (11:20 h−11:40 h) in the electric community, and 3373 ± 2300 lux (09:40 h−10:00 h) in the non-electric community. During the evening (18:00 h–00:00 h), average light intensities, although low, were higher in the electric communities (median electric = 0.168 lux, n = 38; median non-electric = 0.092 lux, n = 43; Mann-Whitney U = 564, p = 0.016; Fig. 4E). In the morning before sunrise (3:00 am–5:20 am), light exposure did not differ between groups (median electric = 0.016 lux, n = 38; median non-electric = 0.014 lux, n = 43; Mann-Whitney U = 672, p = 0.171; Fig. 4F).

During interviews, individuals reported using light (i.e. electric light or solar torches) most commonly in the early evening. In electric communities, 7 individuals had no access to the electric grid (although 1 did report evening electric light exposure 4 days a week), but all 7 used solar torches in the evening (6 of them daily). Of individuals with access to an electric grid, 2 (6.25%) reported not using it during the study period but using solar torches daily, 10 (31.25%) reported some use (1–6 days per week), and 20 (62.5%) reported using electric light every evening. All individuals in electric communities used artificial light during the study period (electric light and/or solar torches), and those with grid access supplemented their electric light use with solar torches. In non-electric communities, 3 (7%) participants did not use any form of artificial light during the study period, 4 (9.3%) reported some evening use (1–6 days per week), and 36 (83.7%) of participants reported using solar torches every evening.

### Nocturnal sleep timing

**Figure 5:**
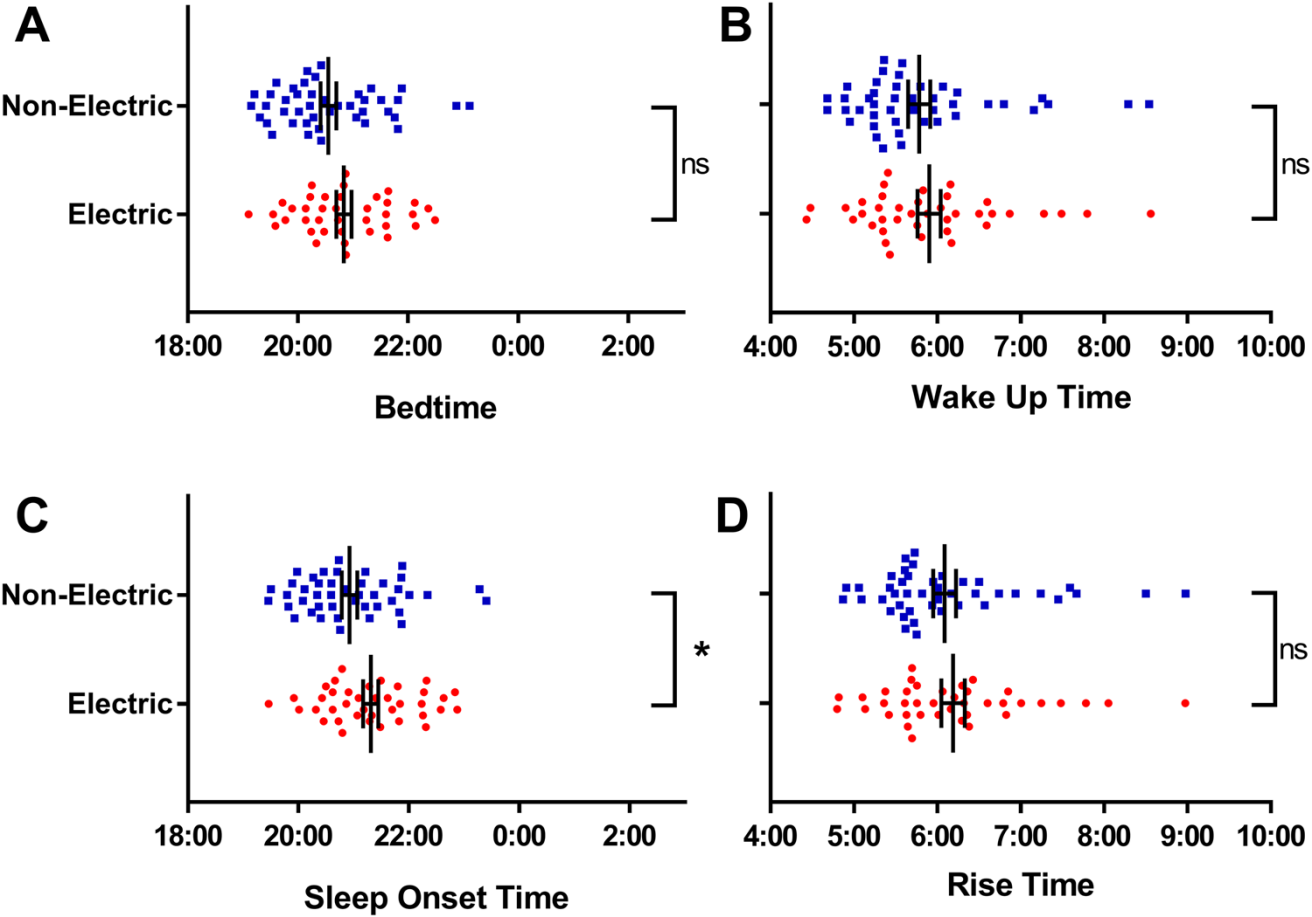
Mean (±standard error) sleep timing by community type. Significant difference between means at p < 0.05 denoted by*.

There was no significant difference between the electric and non-electric communities in Actiwatch measures of average bedtime (*F*_*(2,80)*_ = 2.25, *p* = 0.138), wakeup time (*F*_*(2,81)*_ = 0.54, *p* = 0.467) and rise time (*F*_*(2,81)*_ = 0.39, *p* = 0.533) (Fig. 5A,B,D; Table 2). Sleep onset, however, was significantly delayed in the electric communities (*F*_*(1,80)*_ = 5.50, *p* = 0.022), with individuals falling asleep 23 min later on average compared to non-electric communities (Fig. 5C).

Across communities, there was a main effect of adult type on bedtime (*F*_*(2,80)*_ = 4.65, *p* = 0.013), sleep onset time (*F*_*(2,80)*_ = 5.87, *p* = 0.004), wake up time (*F*_*(2,81)*_ = 9.49, *p* < 0.001) and rise time (*F*_*(2,81)*_ = 10.03, *p* < 0.001). Post hoc tests revealed that, in both electric and non-electric communities, all times were significantly earlier in breastfeeding females compared to males (Table 2). In females without breastfeeding children, sleep timing was intermediate between breastfeeding females and males, but differences were not significant. Individual sleep onset and wake data by adult type are provided in Supplementary Materials (Supplementary Fig. 1).

### Nocturnal sleep duration

**Figure 6:**
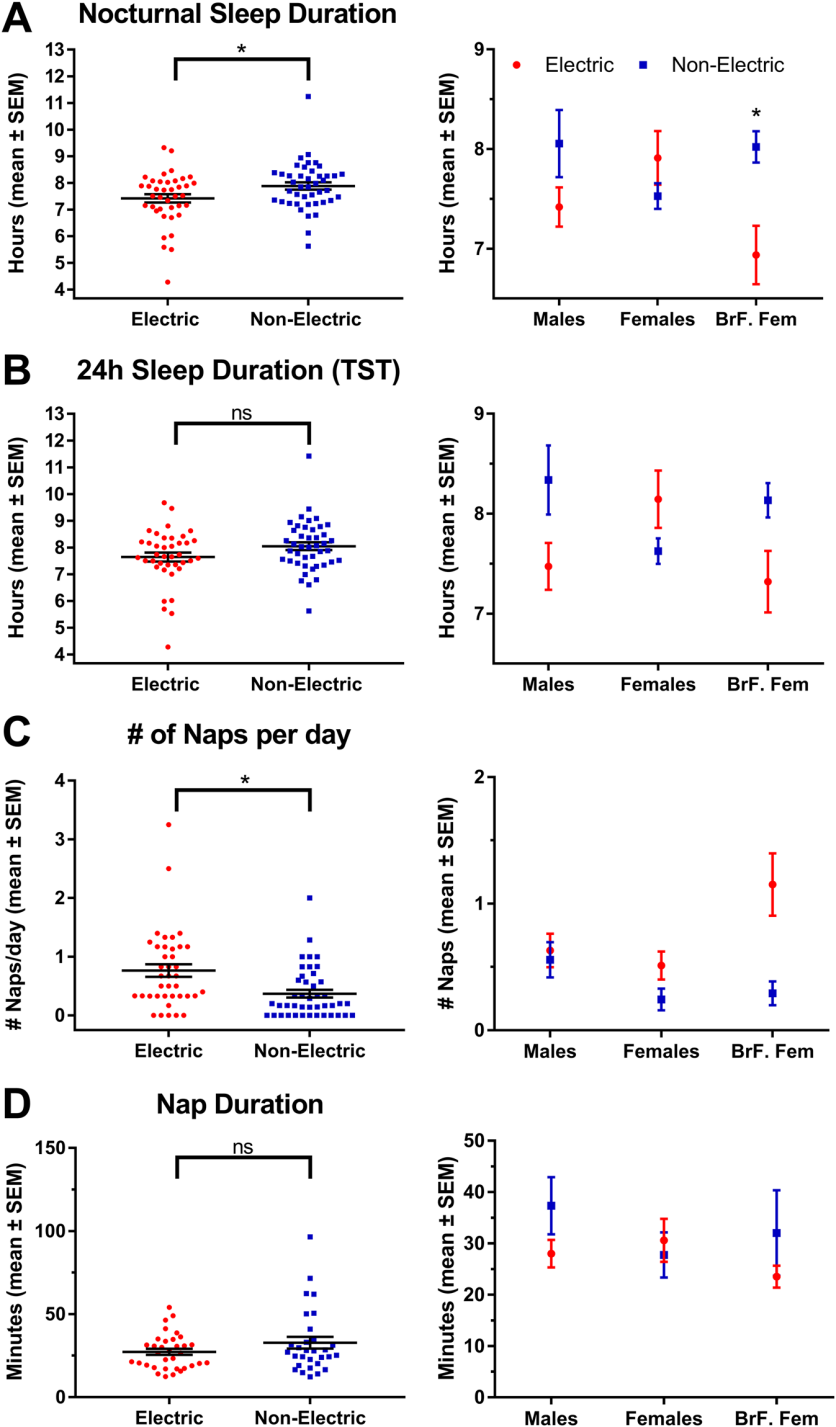
Mean (±standard error) sleep durations by community type and adult type. Panel (A) nocturnal duration; Panel (B) 24 h sleep duration (total sleep time); Panel (C) average number of naps per day; Panel (D) nap duration. Significant difference between means at p < 0.05 denoted by*.

Actiwatch-derived nocturnal sleep duration in adult subjects living in electric and non-electric communities averaged 7.42 ± 0.16 h (445 ± 9 min) and 7.88 ± 0.14 h (473 ± 8 min), respectively (difference = 28 min; Fig. 6A). Two-way ANOVA revealed a significant main effect of community type (*F*_*(2,81)*_ = 4.94, *p* = 0.029), no main effect of adult type (*F*_*(2,81)*_ = 0.55, *p* = 0.58), and a significant interaction between community and adult type (*F*_*(2,81)*_ = 4.60, *p* = 0.013). Tukey’s post hoc tests revealed that community type differences were driven by breastfeeding females with access to electricity, who slept on average 65 min less than did breastfeeding females in the non-electric communities (Fig. 6A). Breastfeeding females in the electric communities slept less than other groups in the electric community, while males and females did not differ significantly in either community. Sleep duration did not correlate with estimates of age (electric *r* = 0.09; non-electric *r* = −0.22; *p*’s > 0.05).

### Nocturnal sleep fragmentation and sleep efficiency

The Actiware 6.0.9 sleep scoring algorithm divided nocturnal sleep into multiple bouts on at least one night in 56% of the electric community sample and 47% of the non-electric sample, for a total of 73 out of 519 nights (Table 3). Among individuals exhibiting sleep fragmentation, the average percentage of nights with fragmentation was 30% in the electric and 20% in the non-electric communities. Prevalence was highest in breastfeeding females in the electric communities.

**Table 3:** Prevalence of sleep fragmentation by percent of sample and percent of total nights of recording.

% of Sample	Males	Females	BrF. Females	Average
Electric	53.9	46.2	69.2	56.4
Non-Electric	25.7	61.5	53.3	46.8
Average	39.8	53.8	61.3	
**% of Nights**	**Males**	**Females**	**BrF. Females**	**Average**
Electric	30.5	26.2	34.7	30.5
Non-Electric	21.4	15.5	23.8	20.2
Average	26.0	20.8	29.2	

Note: Sleep fragmentation was defined as nocturnal bouts of activity detected by actimetry that were sufficient to divide nocturnal sleep into two or more bouts of sleep.

**Figure 7:**
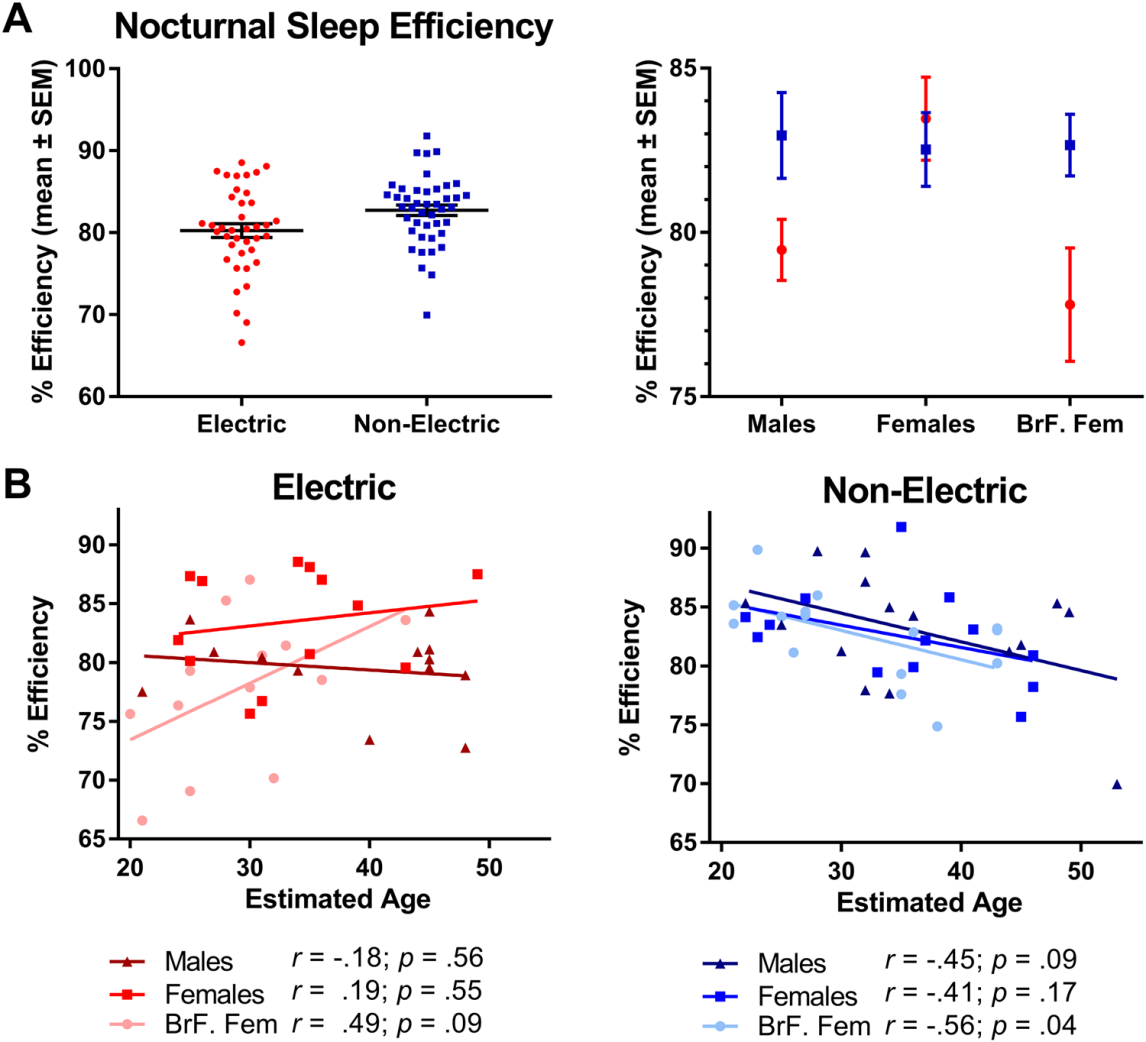
Panel (A) Mean (±standard error) sleep efficiency by community type and adult type. Panel (B) Relationship between sleep efficiency and estimated age (as age & birthdays are not tracked) for adult types within each community.

**Figure 8:**
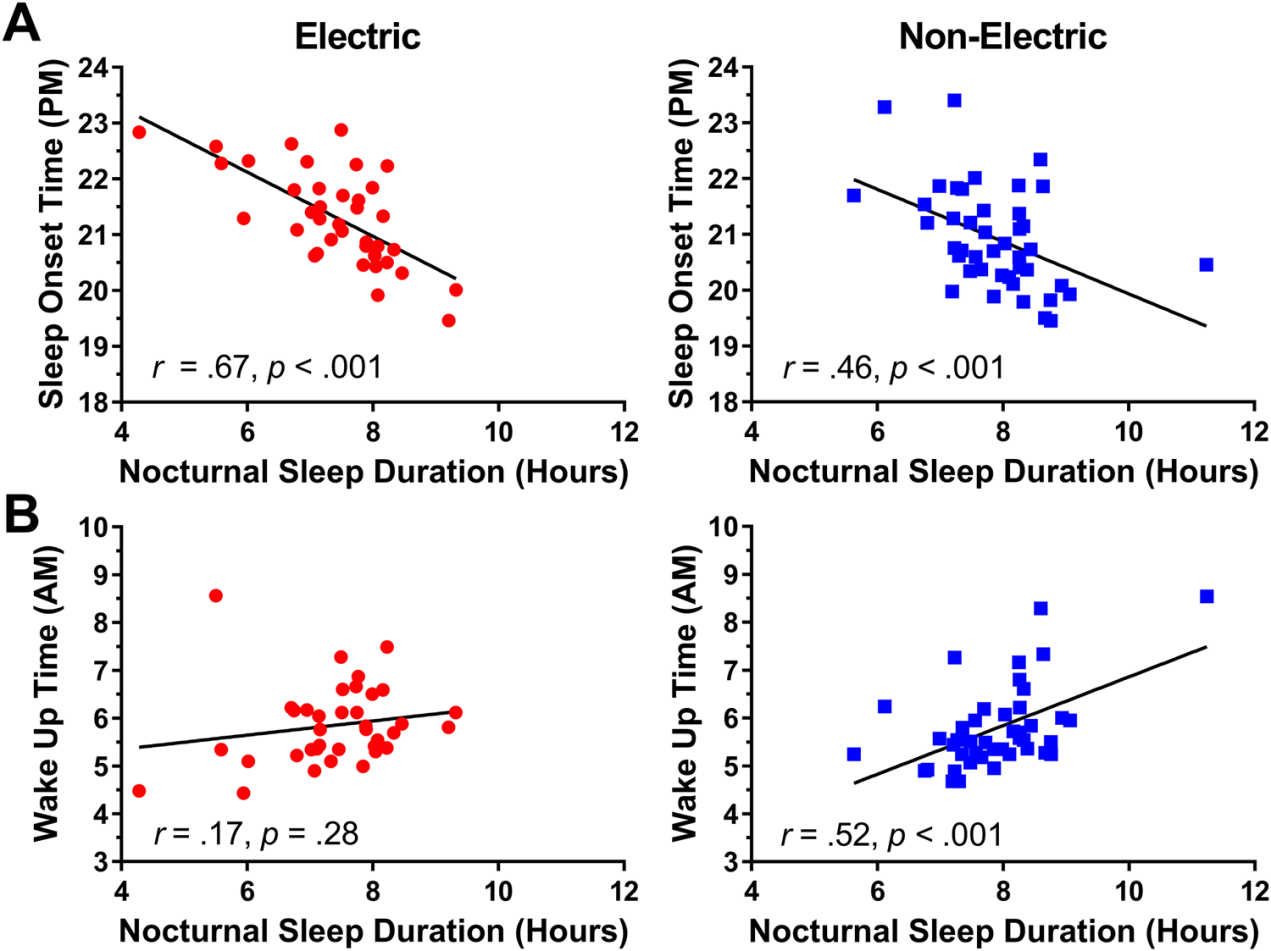
Scatterplots of the relationship between nocturnal sleep duration with sleep onset time (Panel A), and wake onset time (Panel B) in the electric (left panels; red) and non-electric (right panels; blue) communities.

Fragmentation contributed to the nocturnal sleep efficiency score, which was 3.0% lower in the electric communities compared to the non-electric (Table 2; Fig. 7A). An analysis of covariance model was used to test the effects of community type (electric vs non-electric), adult type (males, females, breastfeeding mothers), and the covariate age on sleep efficiency. The analysis showed a statistically significant interaction between the covariate age and community type (*F*_(1,72)_ = 8.32, *p* = 0.005). Sleep efficiency was related to age only in the non-electric community. For this reason, separate tests were conducted for both electric and non-electric communities. In the electric community, there was a main effect of adult type (*F*_(2,35)_ = 4.44, *p* = 0.019), with lower sleep efficiency in males and breastfeeding females compared to females (Table 2; Fig. 7B). In the non-electric community, there was no main effect of adult type (*F*_(2,39)_ = 0.47 *p* = 0.629). Relationships between age and sleep efficiency by adult type were weak for both communities (Fig. 8C,D), and significant only for breastfeeding females in non-electric villages, with sleep efficiency decreasing with age (*F*_(1,39)_ = 10.5, *p* = 0.002).

Subjective reports from interviews indicate that 90.7% of individuals in the electric and 92.3% in the non-electric communities, report waking up during the night. The cause of sleep interruptions in both community types was most frequently attributed to infant care (i.e. babies crying), and dogs barking (Supplementary Fig. 2). Despite this, the majority of individuals (>60%) in each community reported feeling that they slept “enough”.

### Daytime napping and 24 h total sleep time

Actiwatch-derived nap data show that individuals in the electric communities took more naps per day (median electric = 0.67, n = 39; median non-electric = 0.20, n = 43; Mann Whitney *U* = 478.5, *p* = 0.001). The difference was driven primarily by breastfeeding females in the electric communities (Fig. 6C). Nap duration did not differ between community type (median electric = 26.6 min, n = 34; median non-electric = 27.5 min, n = 30; Mann Whitney *U* = 458.5, *p* = 0.49; Fig. 6D).

To determine whether daily naps may compensate for reduced sleep in the electric communities, naps and nocturnal sleep were summed to yield total sleep time (TST; Fig. 7B). In this cumulative measure of sleep, there was no significant main effect of community (electric = 7.65 ± 0.17 h; non-electric = 8.02 ± 0.13 h; *F*_*(1,76)*_ = 3.07, p = 0.084) or adult type (*F*_*(1,76)*_ = 0.23, p = 0.079), but there was a significant interaction (*F*_*(1,76)*_ = 4.68, p = 0.012). As with nocturnal sleep duration, TST in breastfeeding females was lower in electric communities compared to the non-electric communities, although the difference was not significant after correction for multiple comparisons.

### Relationships between sleep timing and duration

Nocturnal sleep onset was delayed and duration shorter in communities with electric lighting. If reduced duration was caused by a delayed sleep onset but fixed wake time, then duration should be negatively correlated with onset time, and weakly or uncorrelated with wake time. In the electric communities, nocturnal sleep duration did exhibit a strong negative correlation with both bedtime (*r* = −0.47, *p* < 0.00) and sleep onset (*r* = −0.42, *p* < 0.00) and no significant correlation with either wake up time (*r* = 0.19, *p* > 0.05) or rise time (*r* = 0.17, *p* > 0.05) (Fig. 8). Removal of one outlier (>3.5 standard deviations from the mean) from the electric community bedtime and sleep onset data did not change this result. In the non-electric communities, nocturnal sleep duration correlated negatively with both bedtime (*r* = −0.49, *p* < 0.00) and sleep onset (*r* = −0.46, *p* < 0.00), and positively with both wake up time (*r* = 0.52, *p* < 0.00) and rise time (*r* = 0.52, *p* < 0.00).

### Environmental variables

**Figure 9:**
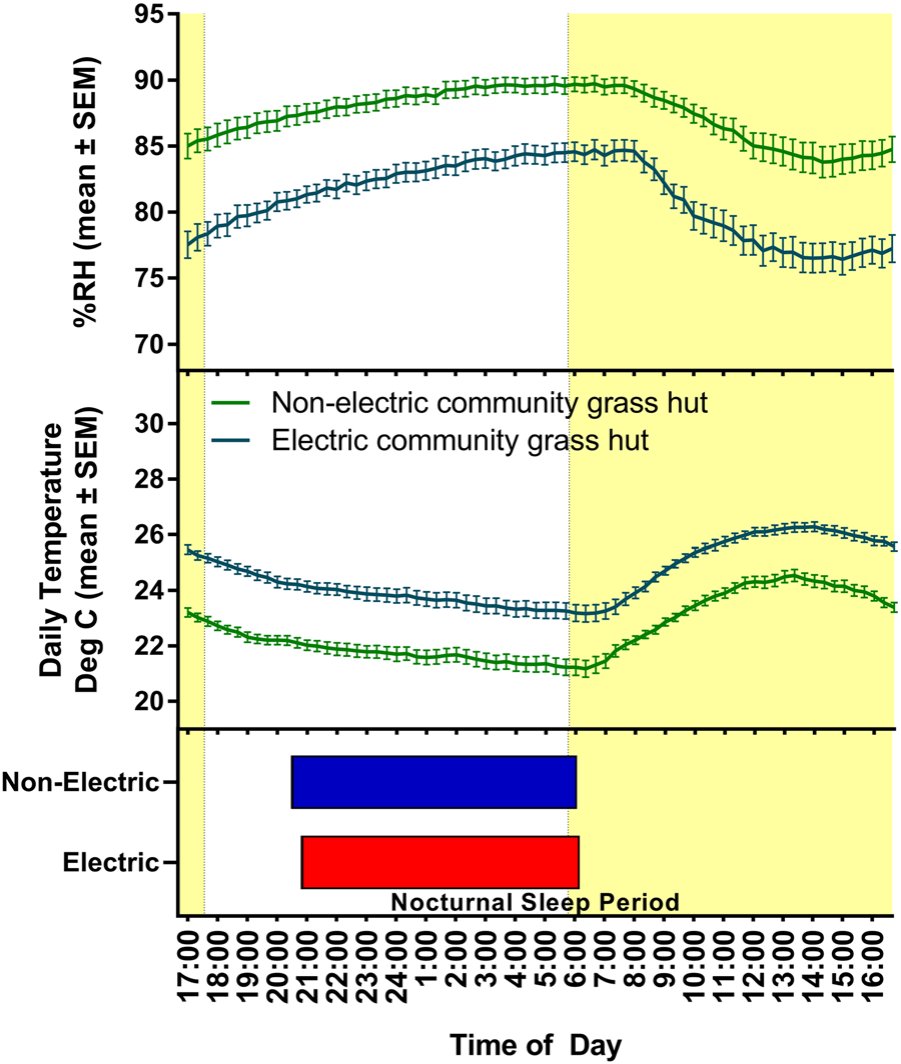
Nocturnal sleep timing in the electric (red bar) and non-electric (blue bar) communities, aligned with daily waveforms of average daily temperature (middle panel; mean degrees Celsius ± standard error) and relative humidity (upper panel; mean % + standard error). Temperature and humidity recorded using iButtons (Maxim Integrated, San Jose, CA) placed in grass huts near sleeping spaces during the study period (April 15–May 7, 2017).

Sleep timing, duration, and efficiency can be affected by environmental stimuli, including light, temperature, humidity, and co-sleepers. Residents in both communities went to bed approximately 3–3.5 h after sunset, which occurred between 17:17 h−17:32 h during the study, and awoke very close to sunrise, which occurred between 05:52 h−06:00 h (Fig. 9). Wake times were on average closer to sunrise than to transitions in ambient temperature and relative humidity, which, at the time of recording, occurred after sunrise, as measured by iButtons in sleeping huts.

A role for evening light exposure in the relationship between sleep onset time and sleep duration is suggested by a significant negative correlation between evening light and nocturnal sleep duration in the electric community (*r* = −0.39, p = 0.02). Evening light was not significantly correlated with sleep duration in the non-electric community (*r* = −0.06, p = 0.70), and morning light exposure was not significantly correlated with sleep duration in either group.

In neither community was sleep efficiency or sleep duration significantly related to average nighttime temperature or humidity (correlation coefficients *r* < 0. 2, *p’*s < 0.05; Supplementary Fig. 3). The absence of a relationship may be due to relatively low variability of average temperature and humidity across the nights of this study. A few of the sleeping huts in villages with electricity were constructed with tin and cement, and these had lower humidity at night compared to grass huts, but the small number of these huts precluded analyses by hut types.

All participants in this study shared sleeping quarters with multiple children or adults. Higher numbers of co-sleepers might be expected to increase the number of nocturnal awakenings, and thereby reduce sleep efficiency and potentially sleep duration, as has been previously reported^30,34,40,41^. In the present study, the average number of co-sleepers was slightly greater in the non-electric communities (4.5 co-sleepers) compared to the electric communities (3.6 co-sleepers), yet the non-electric communities had both longer nocturnal sleep and higher sleep efficiency. Also, neither nocturnal sleep duration nor nocturnal sleep efficiency were significantly correlated with the number of individuals sharing a sleeping space in the electric (duration: *r* = −0.06, *p* = 0.73; efficiency: *r* = −0.09, *p* = 0.58) or the non-electric communities (duration: *r* = −0.01, *p* = 0.96; efficiency: *r* = −0.11, *p* = 0.50). There was also no relationship when correlations were calculated using the number of adult and child co-sleepers separately (Supplementary Fig. 4).

## Discussion

**Figure 10:**
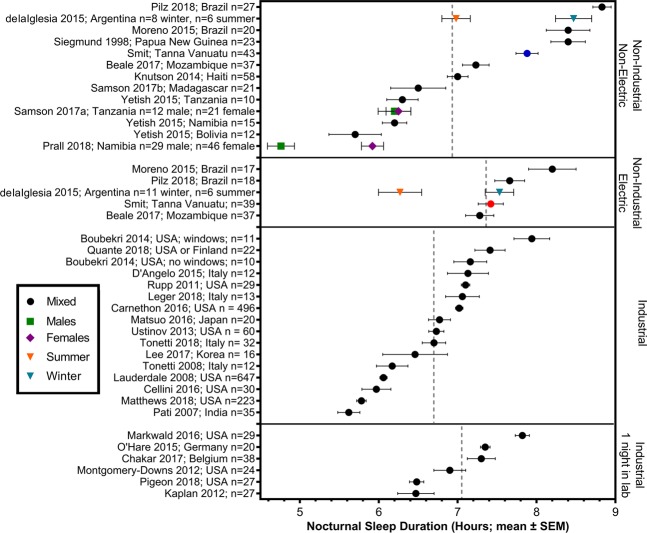
Mean (±standard error) nocturnal sleep duration in hours recorded by actigraphy. Vertical dashed lines represent the mean nocturnal sleep duration for each group. Studies of industrialized societies were included if they used Actiwatch accelerometers (currently Phillips Respironics, Inc.) and associated sleep scoring software. Only data from healthy adults (>18 years of age) on non-restricted sleep schedules were included. If reports included analysis with multiple Actiwatch software settings (e.g. different activity sensitivity thresholds), only the results using default settings were included in the table, to permit comparison with the present study results. Due to a limited number of non-industrial studies done to date, all non-industrial studies were included regardless of recording device. Differences in sleep detection methods, or external factors such as differences in season may contribute to differences in sleep duration.

This is the first actigraphy study of sleep timing and duration in indigenous Melanesians living small scale, traditional horticultural lifestyles in the south pacific island nation of Vanuatu. We found that habitual sleep duration among the Ni-Vanuatu of Tanna Island is long compared to several small-scale hunter-gatherer, agrarian and pastoralist societies in Africa and Bolivia^27–30^, and compared to most samples from industrialized western populations studied by actimetry using Actiwatches (Fig. 10). We also found that nocturnal sleep onset was delayed by 23 minutes and duration was shorter by 28 minutes in participants living in coastal villages with on-demand access to electric light at night. A significant interaction with adult type suggests that the difference in sleep duration is driven primarily by breastfeeding females in the communities with electricity. Reduced nocturnal sleep in this group may have been causally related to increased light exposure during nighttime infant care, compared to breastfeeding females in the non-electric communities who had the same nocturnal responsibilities without on-demand availability of electric light.

We interpret these results as supporting the view that sleep timing and duration in humans is shaped in part by lifestyle adaptations to the opportunities and challenges of particular ecologies. Hunter-gatherer (San, Tsimane and Hadza) and pastoralist (Himba) societies, living at virtually the same latitude as Tanna Island and also studied using wrist-worn actigraphy averaged markedly less daily sleep than the Ni-Vanuatu on Tanna Island^27–30^. Life on Tanna Island is characterized by reliable food access, a mild subtropical climate with relatively low daily and seasonal variability in temperature and day length, absence of predators, and minimal social conflict. Under these conditions, there may be no special fitness advantage of short sleep. Conceivably, there may be a fitness advantage favoring a short sleep genotype in hunter-gatherer and pastoralist societies that is not present in the horticulture-based lifestyle on Tanna Island. Alternatively, short sleep durations in some groups may reflect less favorable sleeping conditions, which could imply that these groups are chronically in sleep deficit. The similar latitude, and thus daylength, sunrise, and sunset times, rule these out as explanations for differences between the Ni-Vanuatu and hunter-gatherer and pastoralist societies studied to date (Supplementary Fig. 5).

We also interpret these results within the context of the *developing economy sleep degradation hypothesis* and the *postindustrial sleep degradation hypothesis*^29^. Although sleep duration on Tanna Island was long by comparison with most actigraphy studies of industrialized western populations (Fig. 10), sleep efficiency was low (80–83%)^42^. This may reflect environmental disturbances, such as having multiple co-sleepers, and housing that offers little protection from surroundings (e.g. noise and weather). Compared to Western homes, the walls of dwellings in Vanuatu are thin and uninsulated and allow greater exposure to outdoor temperatures, and greater sound transmission when wild dogs bark or neighbouring babies cry. Reported sleep disruptions seem to reflect differences in location. For instance, the electric communities are closer to developed roads and reported more automobile related noise disruptions, which would be expected to increase as industrialization progresses. Noise is proposed as a large component of the ‘developing economy sleep degradation hypothesis’ since increasing population density paired with traditional housing offers little buffer^29^. In addition, participants living in villages with electric lighting exhibited delayed and shorter nocturnal sleep. This was associated with increased exposure to evening light, and was particularly prevalent in breastfeeding females, who would be expected to experience more nocturnal waking and evening light exposure. Thus, sleep on Tanna Island exhibits characteristics of developing economy sleep degradation (suggested by low sleep efficiency) and post-industrial sleep degradation (suggested by delayed and reduced nocturnal sleep associated with access to on-demand electric light in the evening). While modern standards of living (more comfortable sleeping arrangements, protection from elements, buffer from noise, etc.) may improve sleep, access to lighting around the clock and other factors (e.g., work or school schedules, daylight saving time, etc.) may counteract some of these improvements.

The difference in average nocturnal sleep duration between the electric and non-electric villages in our study sample was 28 minutes. A reduction of this magnitude in industrialized populations is thought to be physiologically and behaviorally significant, especially if accumulating over days of the work week or longer^14,43^. It is possible that the 7.88 h average nocturnal sleep duration in Tanna Island villages without electricity represents a surfeit, and that a roughly half-hour reduction in villages with electricity is of no functional consequence. If 28 min less sleep at night does represent a deficit, then we might expect to see an effort to compensate by increased daytime napping. While both study groups exhibited some daytime sleep, naps were significantly more prevalent in villages with electricity. When these naps were combined with nocturnal sleep to yield total daily sleep time, the difference in sleep duration between villages with and without electricity was no longer statistically significant. This suggests that daytime naps are at least in part compensatory and that the shorter nocturnal sleep duration in electric communities represents a deficit.

Presumably, the magnitude of differences in sleep timing and duration between communities with and without electric lighting on Tanna Island is limited by continuous exposure to natural light throughout the day in both groups. Morning light opposes the phase delaying effect of evening light^2,3,5^, and increased daytime light decreases sensitivity to artificial evening light^44^. Given the similarity of daytime light exposure patterns in the coastal and inland villages, evidence for a significant effect of on-demand electric light in coastal villages is notable. Another factor that may limit differences between groups is the use of solar torches after sunset in both communities. However, torches provide only low intensity light that is typically directed toward objects and away from the eyes. Despite the use of torches in the non-electric communities, those living in the electric communities showed more light exposure during the first hours of the night (18:00–0:00), and this was associated with delayed sleep onset times and less nocturnal sleep. Delayed sleep onsets may be due to a phase-delay of the circadian pacemaker controlling sleep onset, an acute effect of evening light on alertness (possibility mediated by suppression of melatonin secretion) or both. Additional studies designed to measure circadian phase physiologically (e.g., by serial salivary melatonin sampling) are required to address this. In either case, the results support a hypothesis that exposure to artificial light after sunset can delay sleep onset and reduce sleep duration. Such effects can be expected to increase as access to electricity on Tanna Island expands.

Historical writings have been taken to suggest that in preindustrial western European populations, nocturnal sleep habitually occurred in two bouts - so-called ‘first and second sleep’ - separated by an hour or more of midnight waking^45^. Segmented patterns suggestive of ‘first and second sleep’ have been observed in a small-scale agricultural society in Madagascar^29^. In the present study, 14% of the 519 recorded nights exhibited a bout of nocturnal waking that was sufficient for the state detection algorithm to score two separate bouts of sleep. However, this pattern does not appear to fit with the concept of ‘first and second sleep’ as a reliable sleep phenotype. About 50% of the subjects showed evidence of split sleep, and among those that did, the pattern was sporadic, with split sleep typically evident on only one of up to eight nights of recording. Splits also occurred at variable times of night. Nocturnal sleep in the indigenous residents of Tanna Island is therefore best described as monophasic, with occasional opportunistic daytime naps.

An increase in the number of sleepers sharing the same sleep surface or room can reduce sleep efficiency^30,34,40,41^ or duration^46^. The number of co-sleepers was slightly greater in the non-electric communities, but sleep duration and efficiency were lower in the electric communities. Also, there was no association between the number of co-sleepers and sleep duration and efficiency in either community. In addition, residents of Tanna commonly sleep on hard surfaces, which would not transfer movement between adjacent co-sleepers. For these reasons, it is unlikely that any differences in the number of co-sleepers would account for differences in sleep duration and efficiency between communities.

Decreased sleep efficiency with increased age is a commonly reported association^42^. Although the range of ages was limited in the present sample (adults, estimated ages 20–53 years), a negative relationship with age and sleep efficiency did emerge, but only in non-electric communities. In communities with an electric grid, the lack of a relationship indicated that young adults also exhibited lower sleep efficiency, something that was not apparent in the young adults of the non-electric communities. A non-electric Haitian population showing lower sleep efficiency in younger adults suggests lifestyle factors, such as, increased childcare responsibilities, nocturnal household duties, or engagement in social activities that mask physiological age differences^41^, all of which may be more easily facilitated in Tanna’s electric communities.

On Tanna Island, males are often up late drinking kava, which is an important custom in Vanuatu, as the nakamal, where kava is consumed, is an important gathering place for older men to pass along knowledge and advice to young males in the village. Kava is mildly sedating, but males and non-breastfeeding females pooled separately across communities did not exhibit difference in sleep duration or efficiency, suggesting minimal effect of Kava consumption on sleep. We do not have sufficient information to be able to separate and compare sleep on nights with and without Kava consumption, and any effects might not be detectable by actigraphy.

A limitation of this study is that data were collected only in April and May, during the transition from summer to winter, when daylength averages ~11.5 h. Seasonal variation in sleep timing has been reported in traditional hunter-gatherer societies^27,33^, with daily wake-up time in one study population (the San of Namibia) tracking seasonal changes in the time of the daily minimum of environmental temperature more closely than changes in the time of sunrise^27^. In the present study, temperature recorded in representative sleeping huts showed a daily minimum that occurred on average 26 min after sunrise, in both the coastal and the inland villages. Wake onsets on average were closer to sunrise than to the ambient temperature minimum in both groups. Ambient temperature may be less significant for sleep timing on Tanna Island because nights are milder, the daily temperature range is modest (averaging just under 4 °C), and the transition from decreasing to increasing temperature is gradual. Also, temperature is mild throughout the year and seasonal variation is modest. In this respect, the natural sleep environment on Tanna Island may be more similar to modern built sleep environments, in which temperature changes from day to night are minimized. If ambient temperature plays a role in sleep timing, then we would not expect to see a large effect of seasonal temperature changes on sleep parameters in residents of Tanna Island.

The results of this study indicate that sleep measured by actigraphy in the small-scale traditional society on Tanna Island can be differentiated from sleep in western industrialized samples by relatively long duration and low efficiency. Availability of on-demand electric light appears to have a detectable effect on nocturnal sleep onset and duration, but this effect is likely mitigated by exposure to natural light throughout the day. Actigraphy studies of indigenous Ni-Vanuatu living in industrialized population centers elsewhere in Vanuatu may provide further insight into how lifestyle and industrialization shape sleep.

## Supplementary information

Supplementary Information

## Data Availability

The data sets generated during and/or analyzed during the current study are available from the corresponding author on reasonable request (mistlber@sfu.ca).
