# Supplementary material for: Sleep timing and duration in indigenous villages with and without electric lighting on Tanna Island, Vanuatu
Source: Sci Rep. 2019 Nov 21;9:17278. doi: 10.1038/s41598-019-53635-y (PMC6872597; doi:10.1038/s41598-019-53635-y)

**Supplementary Information**

**SREP-19-27302A**

**Sleep timing and duration in indigenous villages**

**with and without electric lighting on Tanna Island, Vanuatu**

Andrea N. Smit^1^, Tanya Broesch^1^, Jerome M. Siegel^2,3,4^, Ralph E. Mistlberger^1*^

^1^Department of Psychology, Simon Fraser University, Burnaby BC V5A1S6

^2^Department of Psychiatry and Biobehavioral Sciences, University of California,

Los Angeles, Los Angeles, CA 90095, USA

^3^VA Greater Los Angeles Healthcare System, 16111 Plummer Street, Los Angeles, CA 91343

^4^Brain Research Institute, University of California, Los Angeles, Los Angeles, CA 90095, USA

*Corresponding author:

R. Mistlberger, PhD

Department of Psychology, Simon Fraser University

8888 University Drive, Burnaby BC

Canada V5A1S6

604-657-7121

mistlber@sfu.ca

**
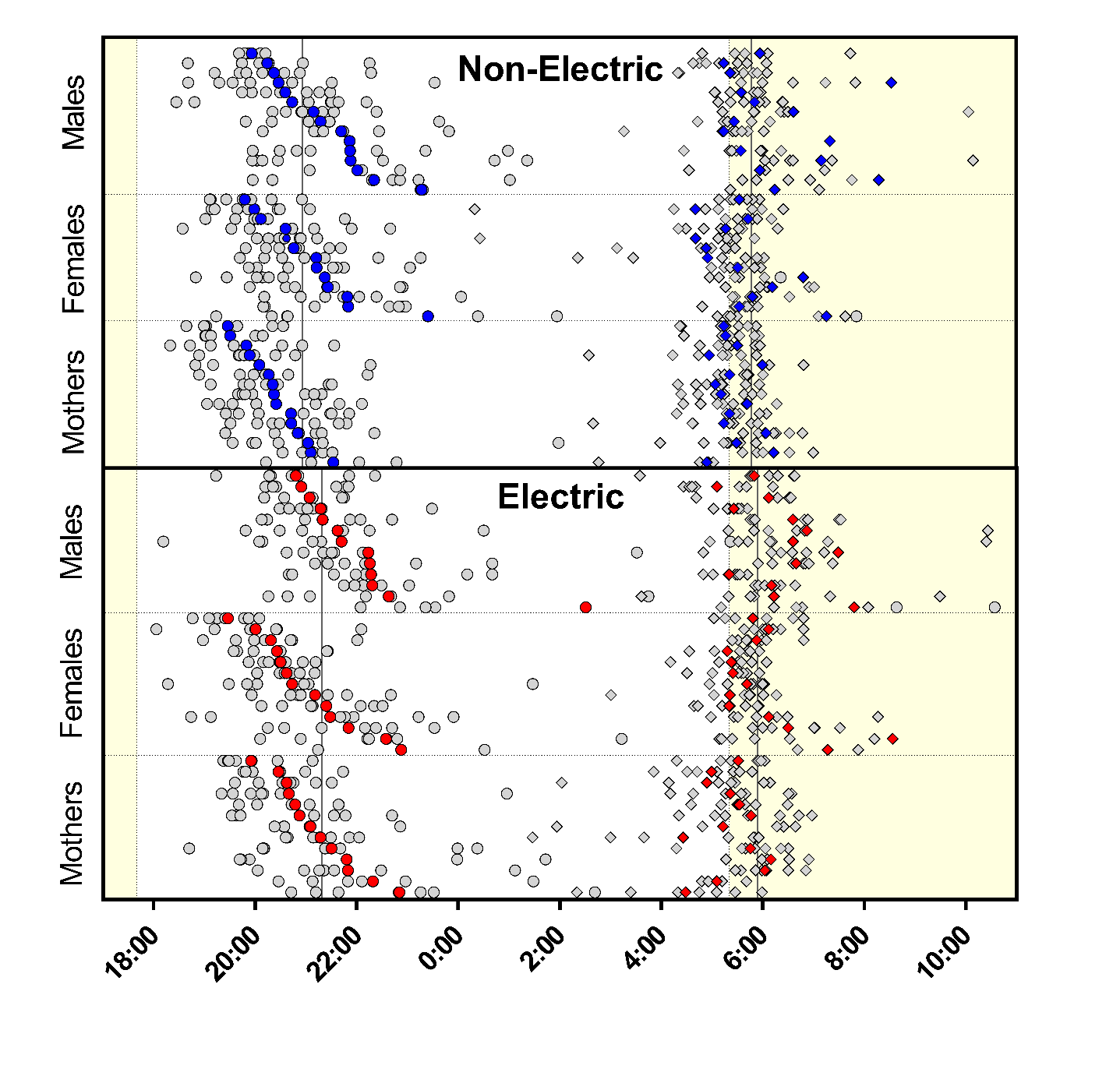
**

## Supplementary Figure 1. Actigraphy-derived daily sleep onset (grey circles) and sleep end (grey diamonds) and the average (coloured marker) for each subject in the non-electric (blue) and electric (red) communities. Vertical lines denote group averages for sleep onset and end. Yellow shading denotes daytime.

##
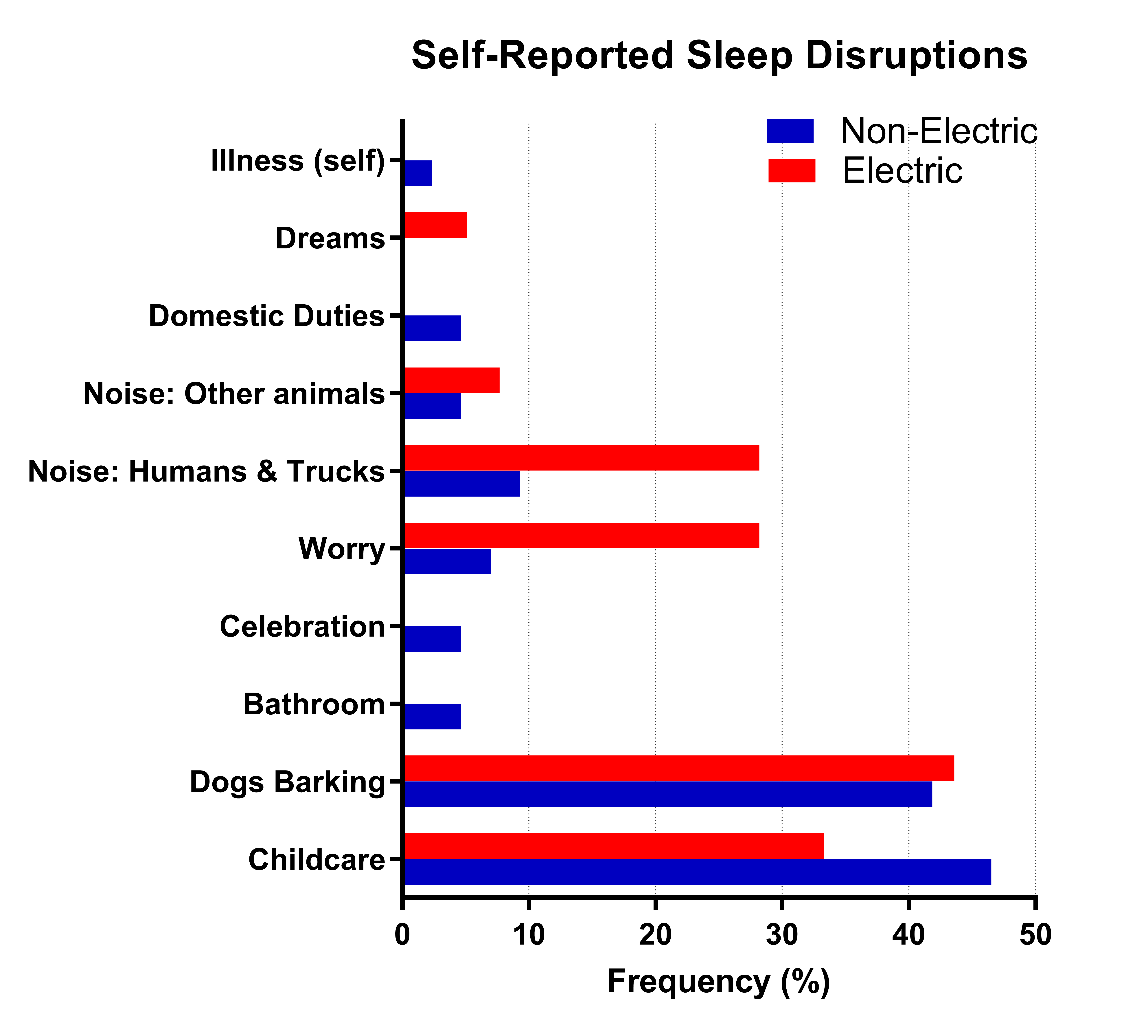


## Supplementary Figure 2. Frequency of self-reported nocturnal sleep disruptions. Domestic duties include preparation of meals and preparing children for school. Human noise includes trucks, music and people’s voices. Childcare includes babies crying, sick, or feeding.

##
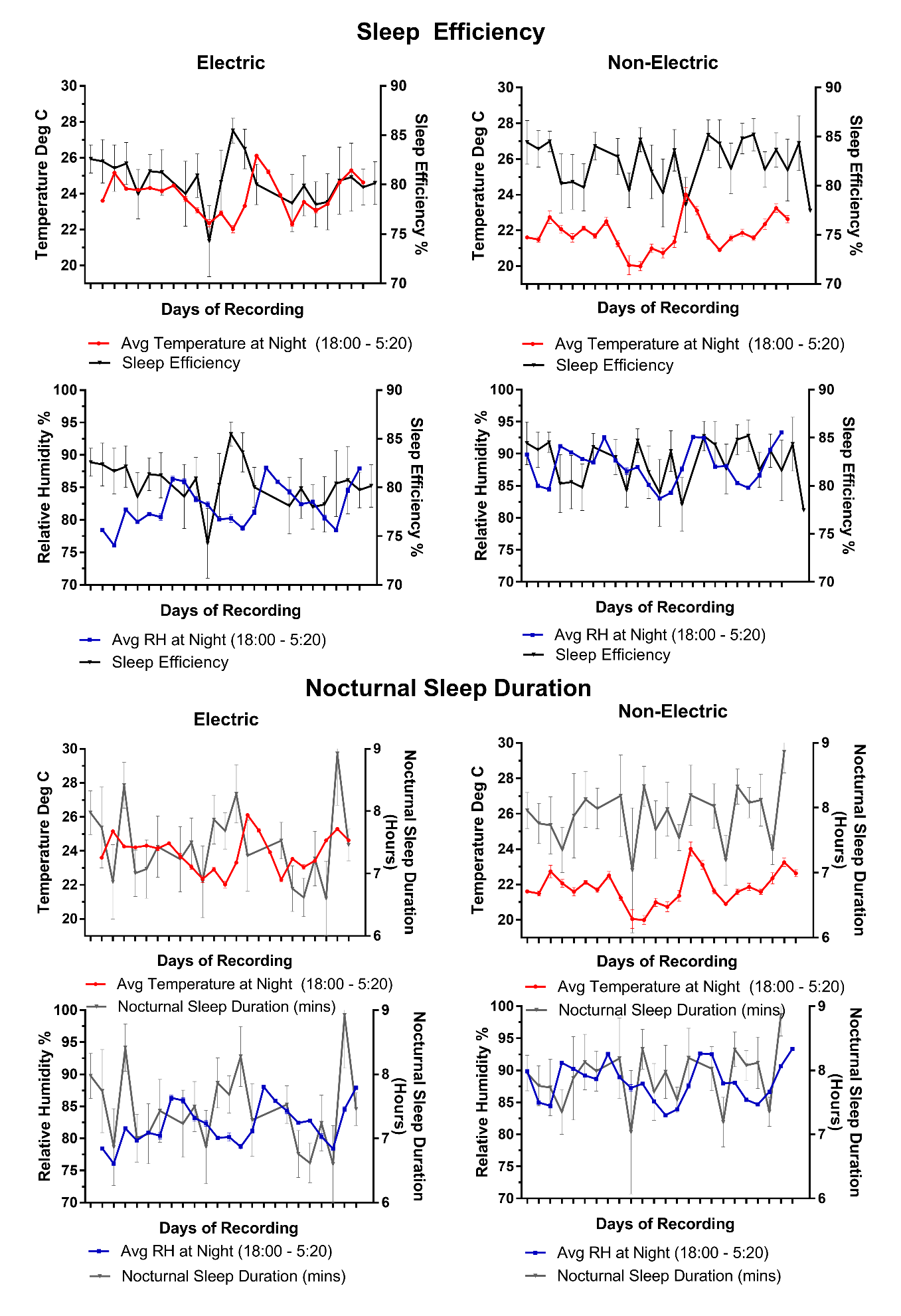


## Supplementary Figure 3. Mean (± standard error) nocturnal sleep efficiency (black curves; upper four panels), minutes of nocturnal sleep duration (grey curves; lower four panels), ambient temperature (red curves) and relative humidity (blue curves) for all nights of the study. Temperature and humidity were recorded using iButtons (Maxim Integrated, San Jose, CA) in grass sleeping huts in the electric (coastal; left panels) and non-electric (inland; right panels) communities.

##
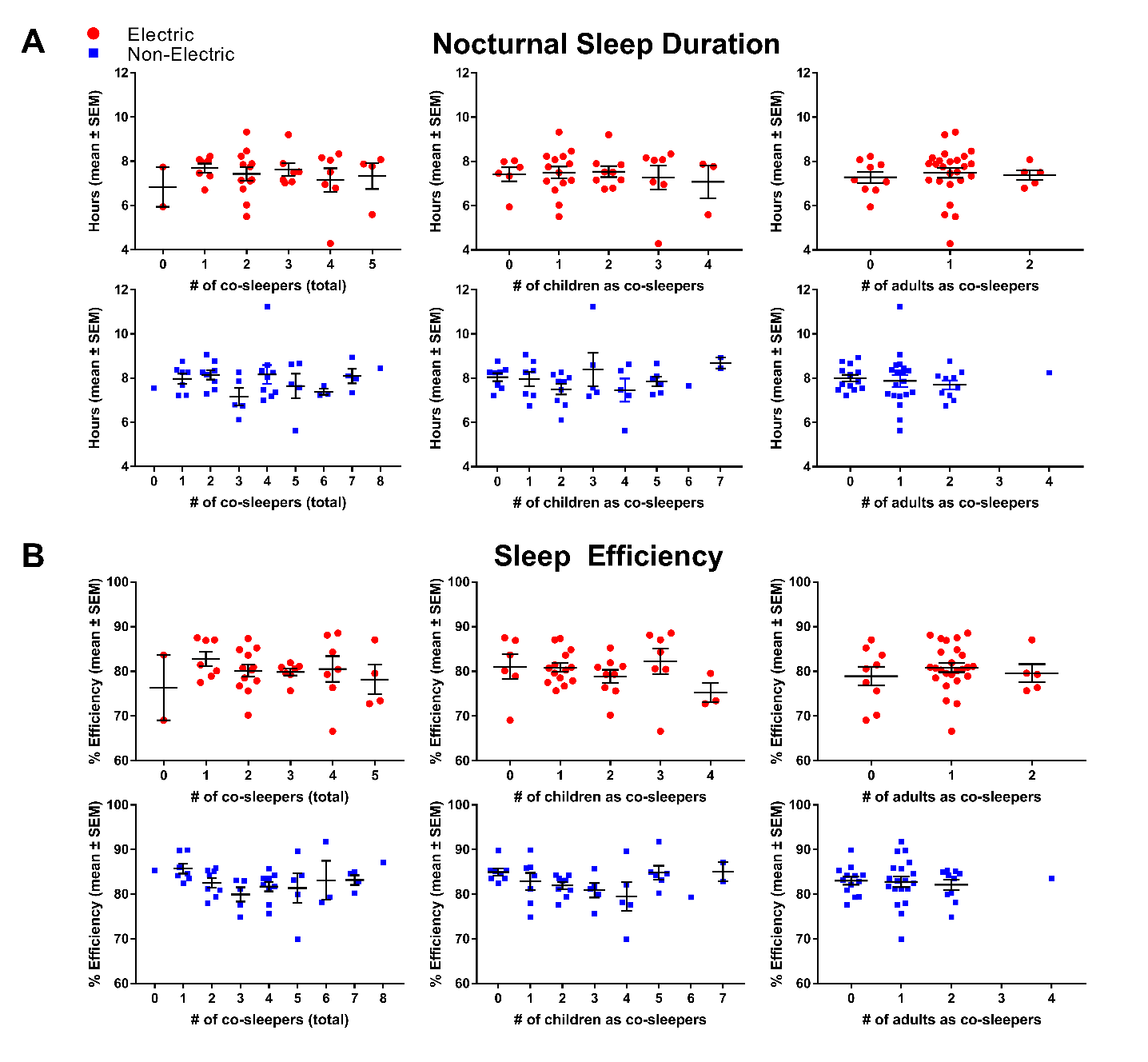


## Supplementary Figure 4. Mean (± standard error) nocturnal sleep duration (Panel A) and sleep efficiency (Panel B) plotted by subjective reports of total number of co-sleepers (including children and adults; left panels), number of children as co-sleepers (middle panels), and number of adults as co-sleepers (right panels) for electric (red) and non-electric (blue) communities.

##
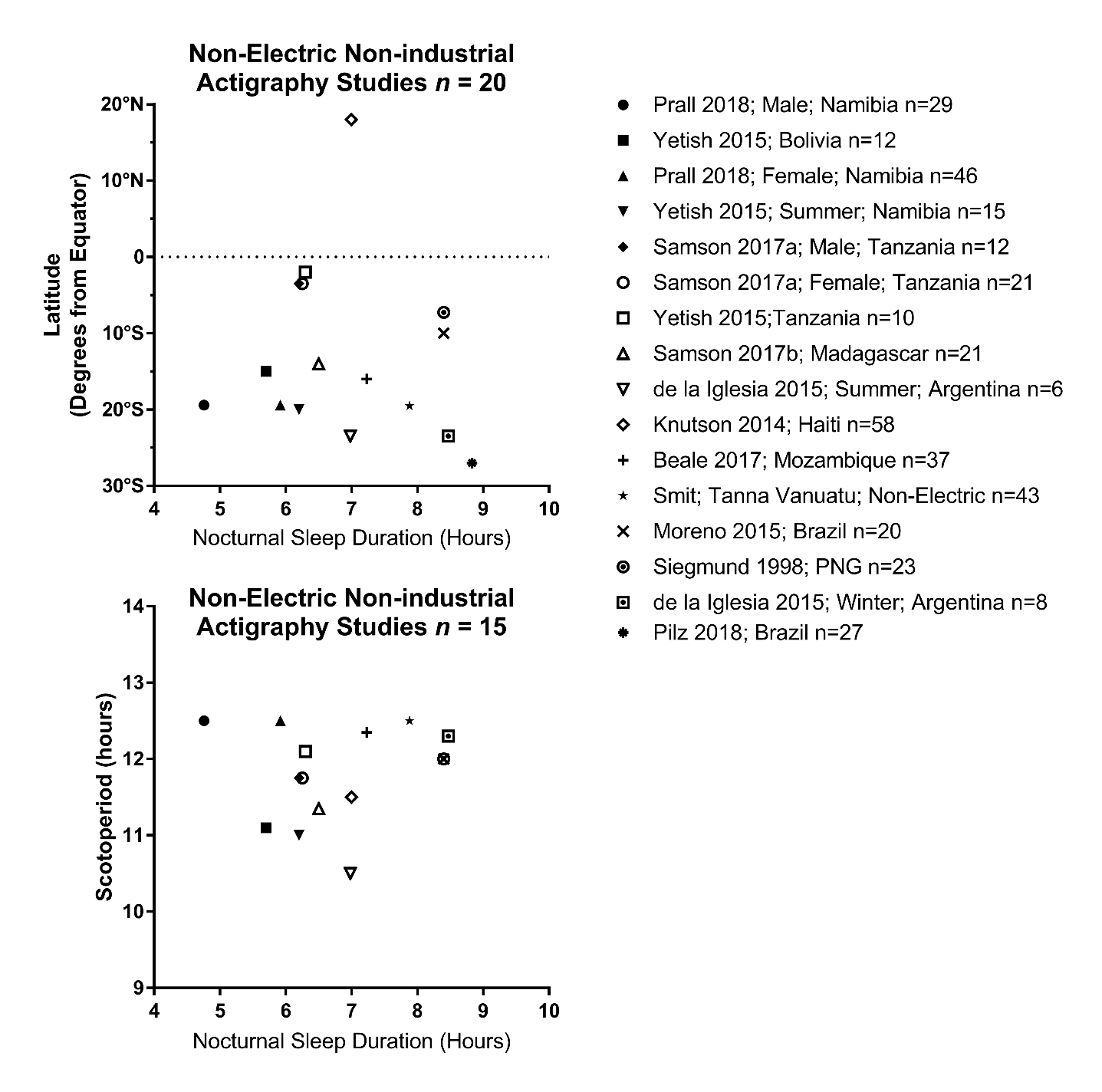


## Supplementary Figure 5. Relationship between nocturnal sleep duration with latitude (upper) and scotoperiod (night length; lower) in recent studies of sleep in non-industrial populations without electricity. Similarities in latitude and night length can be ruled out as independently explaining differences in sleep duration.

**Supplementary References (Figure 5)**

Beale, A. D., Pedrazzoli, M., Bruno da Silva, B. G., Beijamini, F., Duarte, N. E., Egan, K. J., ... & Roden, L. C. (2017). Comparison between an African town and a neighbouring village shows delayed, but not decreased, sleep during the early stages of urbanisation. Scientific reports, 7(1), 5697.

de la Iglesia, H. O., Fernández-Duque, E., Golombek, D. A., Lanza, N., Duffy, J. F., Czeisler, C. A., & Valeggia, C. R. (2015). Access to electric light is associated with shorter sleep duration in a traditionally hunter-gatherer community. Journal of biological rhythms, 30, 342-350.

Knutson, K. L. (2014). Sleep duration, quality, and timing and their associations with age in a community without electricity in Haiti. American Journal of Human Biology, 26, 80-86.

Moreno, C. R., Vasconcelos, S., Marqueze, E. C., Lowden, A., Middleton, B., Fischer, F. M., ... & Skene, D. J. (2015). Sleep patterns in Amazon rubber tappers with and without electric light at home. Scientific Reports, 5, 14074.

Pilz, L. K., Levandovski, R., Oliveira, M. A., Hidalgo, M. P., & Roenneberg, T. (2018). Sleep and light exposure across different levels of urbanisation in Brazilian communities. Scientific Reports, 8(1), 11389.

Prall, S. P., Yetish, G., Scelza, B. A., & Siegel, J. M. (2018). The influence of age-and sex-specific labor demands on sleep in Namibian agropastoralists. Sleep health, 4(6), 500-508.

Samson, D. R., Crittenden, A. N., Mabulla, I. A., Mabulla, A. Z., & Nunn, C. L. (2017b). Hadza sleep biology: Evidence for flexible sleep‐wake patterns in hunter‐gatherers. American journal of physical anthropology, 162(3), 573-582.

Samson, D. R., Manus, M. B., Krystal, A. D., Fakir, E., Yu, J. J., & Nunn, C. L. (2017a). Segmented sleep in a nonelectric, small‐scale agricultural society in Madagascar. American Journal of Human Biology, 29(4), e22979.

Siegmund, R., Tittel, M., & Schiefenhövel, W. (1998). Activity monitoring of the inhabitants in Tauwema, a traditional Melanesian village: rest/activity behaviour of Trobriand islanders (Papua New Guinea). Biological rhythm research, 29(1), 49-59.

Yetish, G., Kaplan, H., Gurven, M., Wood, B., Pontzer, H., Manger, P. R., ... & Siegel, J. M. (2015). Natural sleep and its seasonal variations in three pre-industrial societies. Current Biology, 25(21), 2862-2868.

## Supplementary Material: Interviews.

## Participants were visited during data collection week to complete an interview about lifestyle habits and use of electric light, and again on the last day of data collection (day 7) to completed a follow-up interview, where they indicated if there was anything that had disrupted their sleep from its usual pattern (e.g., illness, celebration, etc.). The interviews were administered by trained local translators and were conducted in the indigenous language specific to each village. This information was used primarily to inform Actiwatch data cleaning (e.g., exclusion of nights in which a participant was sick), and to qualitatively understand aspects of local lifestyles that disrupt sleep.

##
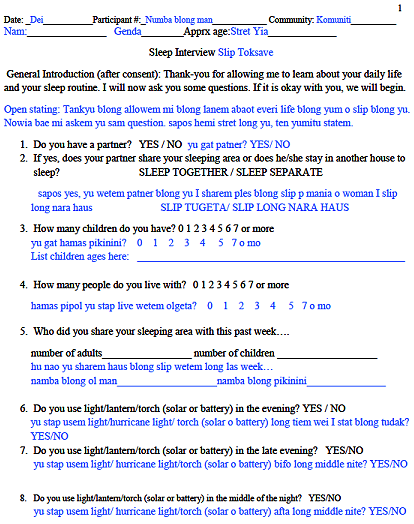


##
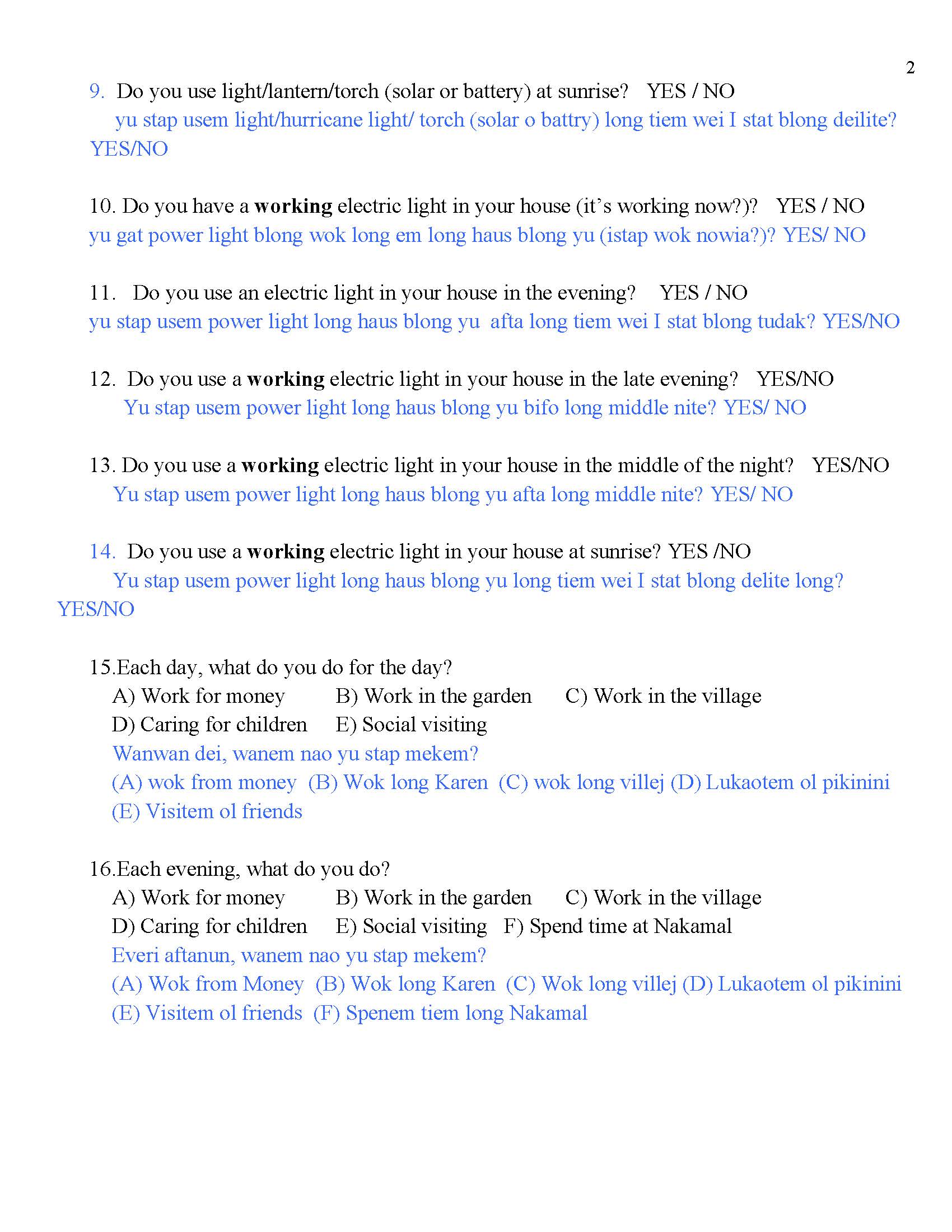

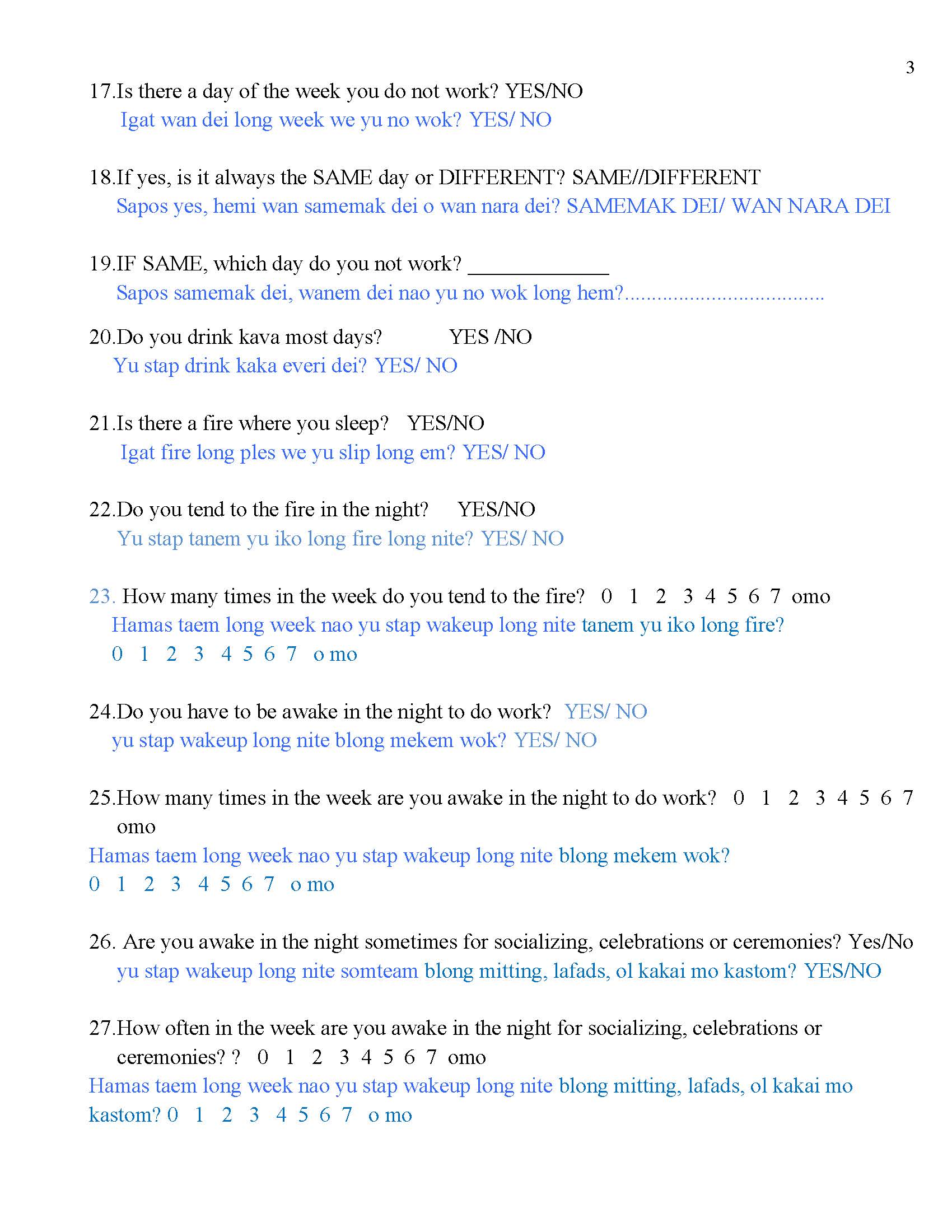

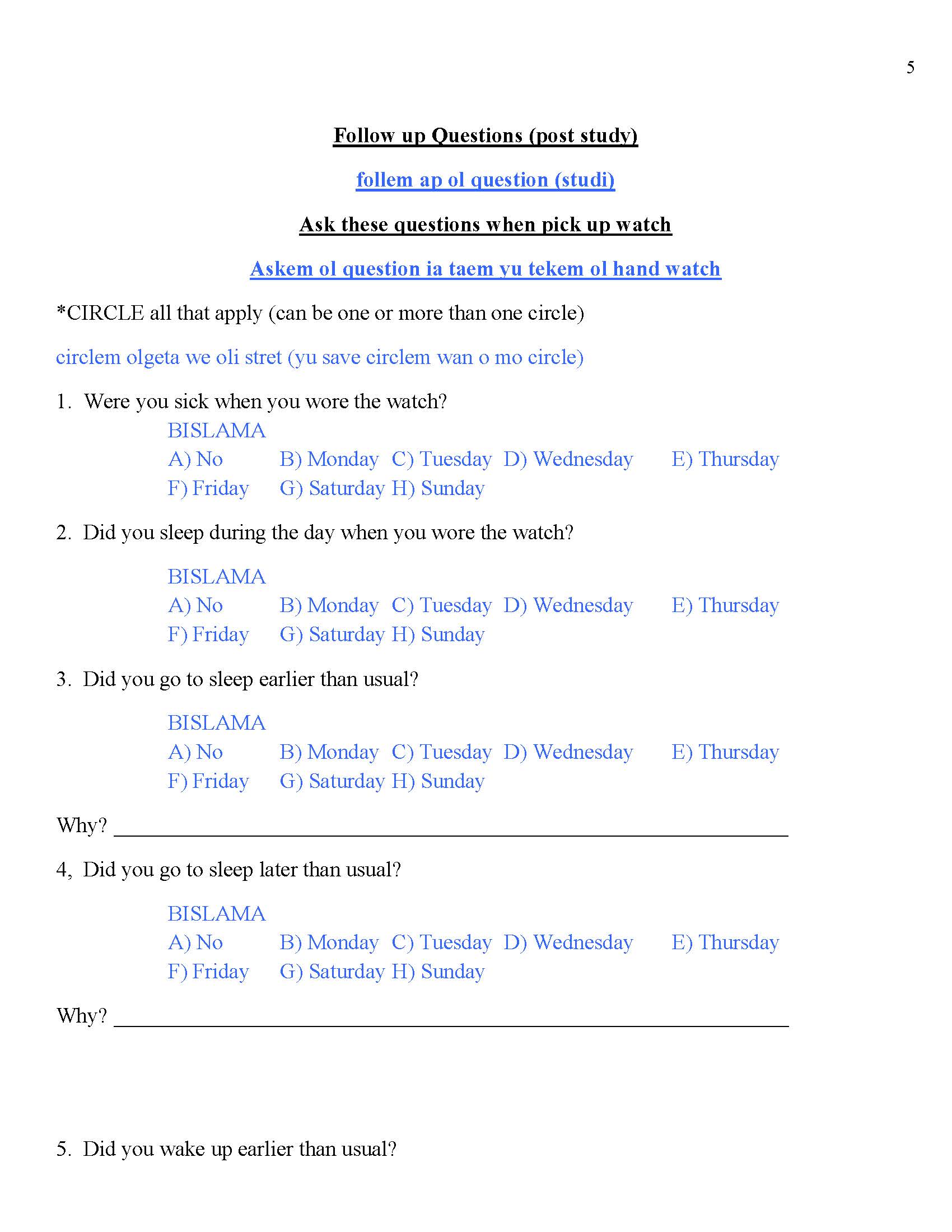

Supplement: Supplementary file 1 — Supplementary Information [file 41598_2019_53635_MOESM1_ESM.docx]
